# Quantum circuit optimization using quantum Karnaugh map

**DOI:** 10.1038/s41598-020-72469-7

**Published:** 2020-09-24

**Authors:** J.-H. Bae, Paul M. Alsing, Doyeol Ahn, Warner A. Miller

**Affiliations:** 1 Department of Electrical and Computer Engineering, University of Seoul, 163 Seoulsiripdae-ro, Tongdaimoon-gu, Seoul, 02504 South Korea; 2 Air Force Research Laboratory, Information Directorate, Rome, NY 13441 USA; 3 Department of Physics, Florida Atlantic University, Boca Raton, FL 33431 USA; 4 Peta Lux Inc., 12 Yanghyeon-ro 405 beon-gill, Seongnam, Gyeonggi-do 13438 South Korea

**Keywords:** Mathematics and computing, Physics

## Abstract

Every quantum algorithm is represented by set of quantum circuits. Any optimization scheme for a quantum algorithm and quantum computation is very important especially in the arena of quantum computation with limited number of qubit resources. Major obstacle to this goal is the large number of elemental quantum gates to build even small quantum circuits. Here, we propose and demonstrate a general technique that significantly reduces the number of elemental gates to build quantum circuits. This is impactful for the design of quantum circuits, and we show below this could reduce the number of gates by 60% and 46% for the four- and five-qubit Toffoli gates, two key quantum circuits, respectively, as compared with simplest known decomposition. Reduced circuit complexity often goes hand-in-hand with higher efficiency and bandwidth. The quantum circuit optimization technique proposed in this work would provide a significant step forward in the optimization of quantum circuits and quantum algorithms, and has the potential for wider application in quantum computation.

## Introduction

In quantum information science there has been significant effort directed towards various physical implementations of quantum bits and quantum circuits^1–11^. The efficient design of quantum circuits for processing quantum information is a fundamental problem in quantum algorithm design and quantum computation because qubits are very expensive resources^12–22^. This is especially important in the regime of quantum computation with limited number of qubits^13–22^. More recent work^23–30^ includes more fundamental quantum information theoretic aspects on quantum computations in relation to the previously mentioned queries. One approach to address this quantum design problem is to adapt some successful approaches that were used in the classical design of circuits. During the development of microelectronics, the separation of device technology and the systems by means of an invariant interface to simplify the design was an essential and outstanding step to cope with the complexity of the system^31^. It is almost certain that this principle of hierarchical design or a related one will also be valid for quantum architecture. Nonetheless, an equivalent invariant interface for the efficient design of quantum circuits is still lacking to the best of our knowledge. In the case of the conventional logic design there is an efficient method called the Karnaugh map^32^. However, applying this method to simplify quantum circuits is nontrivial because the representation of the quantum state evolution in Hilbert space by classical Boolean algebra through Karnaugh map is not quite straightforward^33,34^. Here, we propose a quantum mechanical version of Karnaugh map called the quantum Karnaugh map (QKM) which operates on the Hilbert space state vectors to facilitate the efficient design of universal quantum circuits. Our preliminary study shows an almost 60% and 46% reduction of the number of circuit elements for the four- and five-qubit Toffoli gates, respectively. While the representative, though non-trivial example of the Toffoli gate is simple to demonstrate the implementation of the QKM, realistic algorithm can have much more complexity and number of gates.

**Figure 1:**
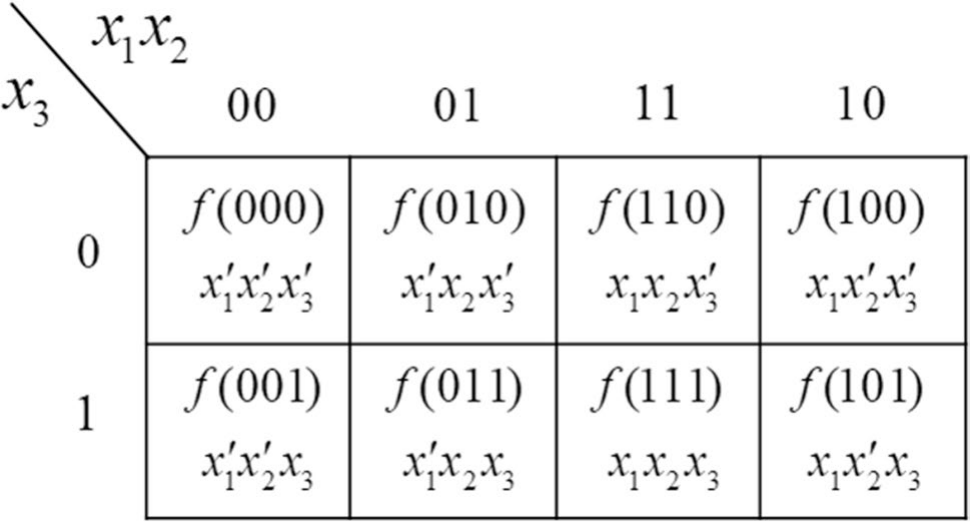
Depiction of a classical Karnaugh map.

In classical logic gate design, the logic functions expressed by the minterm expansion can be generally simplified by utilizing theorems of Boolean algebra such as the consensus theorem:$XY + X^{\prime}Z + YZ = XY + X^{\prime}Z$^32,35^ Here $X,\;Y,\;Z$ are Boolean variables and $X^{\prime},\;Y^{\prime},\;Z^{\prime}$ are their complement forms. The minterm^35^ of $n$ variables is a product of $n$ literals in which each variable appears only once in true or complemented form, but not both. A literal is a Boolean variable or its complement. If we denote the value and the minterm of the truth table of ith row as $a_{i}$ and $m_{i}$, the minterm expansion of logic function f is given by.

1 $$f = \sum\limits_{i} {a_{i} m_{i} } ,$$

where $a_{i} \;{\text{ in }}\;[0,\;1]$. The Karnaugh map^32,35^ is a technique to find a minimum sum-of-products expression for a logic function. A minimum sum-of-products expression is defined as a sum of product terms that (a) has a minimum number or terms and (b) of all those expressions which have the same minimum number of terms has a minimum number of literals. Just like a truth table, the Karnaugh map of a function specifies the value of the function for every combination of the value of the independent variables. A three-variable Karnaugh map is shown in Fig. 1. In the upper row, the Boolean variable $x_{1} x_{2}$ are labeled in the sequence $00,\;01,\;11,\;10$ so that values in adjacent columns differ in only one variable. Each square of the map corresponds to the values of Boolean variables and a minterm as indicated. Minterms in adjacent squares of the map can be combined since they differ in only one variable, e.g. if $f(ijk) = 0$ except for $f(110)$ and $f(100)$, then $x_{1} x_{2} x_{3} ^{\prime}$ and $x_{1} x_{2} ^{\prime}x_{3} ^{\prime}$ combine to form $x_{1} x_{3} ^{\prime}$.

In order to develop analogous quantum Karunaugh map (QKM), we start by recalling a controlled unitary gate $C^{1} (U)$ defined in the $\left\{ {\left| {00} \right\rangle ,\left| {01} \right\rangle ,\left| {10} \right\rangle ,\left| {11} \right\rangle } \right\}$ basis that satisfies the following switching function properties:

2 $$C^{1} (U)\left| {xy} \right\rangle = \left\{ {\begin{array}{*{20}l} {\left| {xy} \right\rangle } & {\quad {\text{ for }}x = 0,} \\ {u_{0y} \left| {x0} \right\rangle + u_{1y} \left| {x1} \right\rangle { = }\sum\limits_{{k = \{ 0,1\} }} {\left| {xk} \right\rangle } \,u_{ky} } & {\quad {\text{ for }}x = 1.} \\ \end{array} } \right.$$

Here, $U_{ij} ,\; \, i,\;j = 0,\;1$ are the unitary matrix elements of $C^{1} (U)$. We found that, instead of counting the cases for the $\left\{ {\left| {00} \right\rangle ,\left| {01} \right\rangle ,\left| {10} \right\rangle ,\left| {11} \right\rangle } \right\}$ basis separately, we obtain the same results by employing an compact 2-qubit basis $\left\{ {\left| {\tilde{0}} \right\rangle_{2} ,\;\left| {\tilde{1}} \right\rangle_{2} } \right\}$ which are defined by.

3 $$\left| {\tilde{0}} \right\rangle_{2} \equiv \left| 0 \right\rangle \otimes I = \left( {\begin{array}{*{20}l} 1 & {\quad 0} \\ 0 & {\quad 1} \\ 0 & {\quad 0} \\ 0 & {\quad 0} \\ \end{array} } \right) = \left( {\begin{array}{*{20}l} I \\ O \\ \end{array} } \right)\quad {\text{and}}\quad \left| {\tilde{1}} \right\rangle_{2} \equiv \left| 1 \right\rangle \otimes I = \left( {\begin{array}{*{20}l} 0 & {\quad 0} \\ 0 & {\quad 0} \\ 1 & {\quad 0} \\ 0 & {\quad 1} \\ \end{array} } \right) = \left( {\begin{array}{*{20}l} O \\ I \\ \end{array} } \right).$$

Here, for simplicity, we denote *I* and *O* for the identity and null $2 \times 2$ matrices in 2-dimensional Hilbert space, respectively. In this compact 2-qubit notation, the first and the second column of $\left| {\tilde{0}} \right\rangle_{2}$ corresponds to two qubit states $\left| {00} \right\rangle$ and $\left| {01} \right\rangle$, respectively. Likewise, the first and the second column of $\left| {\tilde{1}} \right\rangle_{2}$ corresponds to the two qubit states $\left| {10} \right\rangle$ and $\left| {11} \right\rangle$; respectively. One can expand $C^{1} (U)$ in the compact qubit basis as follows.

4 $$C^{1} (U) = C^{1} (U)\left| {\tilde{0}} \right\rangle_{2} \left\langle {\tilde{0}} \right| + C^{1} (U)\left| {\tilde{1}} \right\rangle_{2} \left\langle {\tilde{1}} \right| = \left( {\begin{array}{*{20}l} I & {\quad O} \\ O & {\quad U} \\ \end{array} } \right),$$

where $$U = \left( {\begin{array}{*{20}l} {u_{00} } & {u_{01} } \\ {u{}_{10}} & {u_{11} } \\ \end{array} } \right)$$. The detailed mathematical description of compact qubits and analysis of quantum circuits using QKM is given in the method section and the supplementary information (SI).

## Results

### Decomposition of four- and five-qubit Toffoli Gates

**Figure 2:**
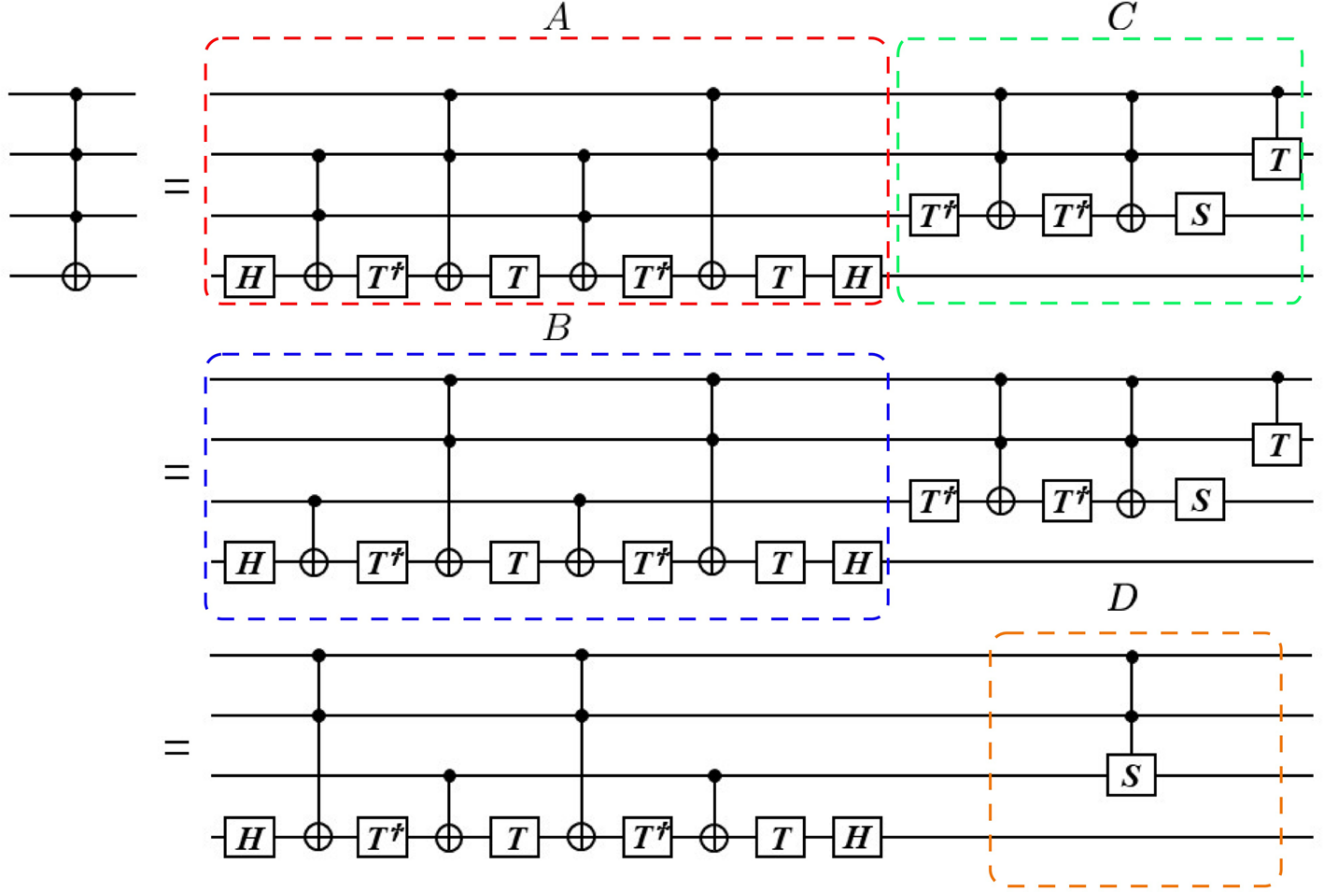
Three equivalent quantum circuit decomposition of a 4-qubit Toffoli gate $C^{3} (X)$. The minimum gate representation of $C^{3} (X)$ is shown in the bottom of the figure. The partial circuits A, B, C and D are explored in Figs. 3 and 4.

One can decompose the given gate in terms of single qubit gates and CNOT gates. The CNOT gate is denoted as the $C^{1} (X)$ gate in this work by substituting *U* by *X* in Eq. (2), where $X$ is a Pauli matrix, $$X = \left( {\begin{array}{*{20}l} 0 & {\quad 1} \\ 1 & {\quad 0} \\ \end{array} } \right)$$. For example, Fig. 2 shows the canonical 4 qubit Toffoli gate at the top of the figure, and its minimum gate representation at the bottom of the figure. The basic single qubit gates used to decompose Toffoli gate are as follows^36^:

5 $$H = \frac{1}{\sqrt 2 }\left( {\begin{array}{*{20}l} 1 & {\quad 1} \\ 1 & {\quad - 1} \\ \end{array} } \right), \, S = \left( {\begin{array}{*{20}l} 1 & {\quad 0} \\ 0 & {\quad i} \\ \end{array} } \right), \, T = \left( {\begin{array}{*{20}l} 1 & {\quad 0} \\ 0 & {\quad e^{i\pi /4} } \\ \end{array} } \right), \, T^{\dag } = \left( {\begin{array}{*{20}l} 1 & {\quad 0} \\ 0 & {\quad e^{ - i\pi /4} } \\ \end{array} } \right).$$

Here, $H$ is the Hadamard gate, $S$ the phase gate and $T$ is the $\pi /8$ gate^29^. These are the elementary single qubit gates acting on the single-qubit state. It was shown that these unitary operations on one qubit and the CNOT gate is sufficient for general quantum programming^12,37–39^. The top quantum circuit is the extension of the Toffoli gate described by the figure 4.9 of Nilsen and Chuang^38^ by adding one more input qubits. The number of elementary gates to construct 4-qubit Toffoli gate $C^{3} (X)$ in the top circuits is 106 which consists of 36 CNOT gates and 70 single qubit gates. The bottom of Fig. 2 shows that the reduced gate has 11 elements which are equivalent to 16 CNOT gates and 29 single qubit gates. This is an almost 60% reduction of the number of elementary gates to implement 4-qubit Toffoli gate. Details of the 4-qubit Toffoli gate $C^{3} (X)$ reduction is given in the Section 3 of SI. The partial circuits A, B, C and D will be explored later in this section and in the SI. The first step of the reduction is replacing a half of the $C^{2} (X)$ gates by $C^{1} (X)$ gates. Here $C^{2} (X)$ gate is the 3-qubit Toffoli gate shown in Fig. S2 of SI and is described by equation (S3).

**Figure 3:**
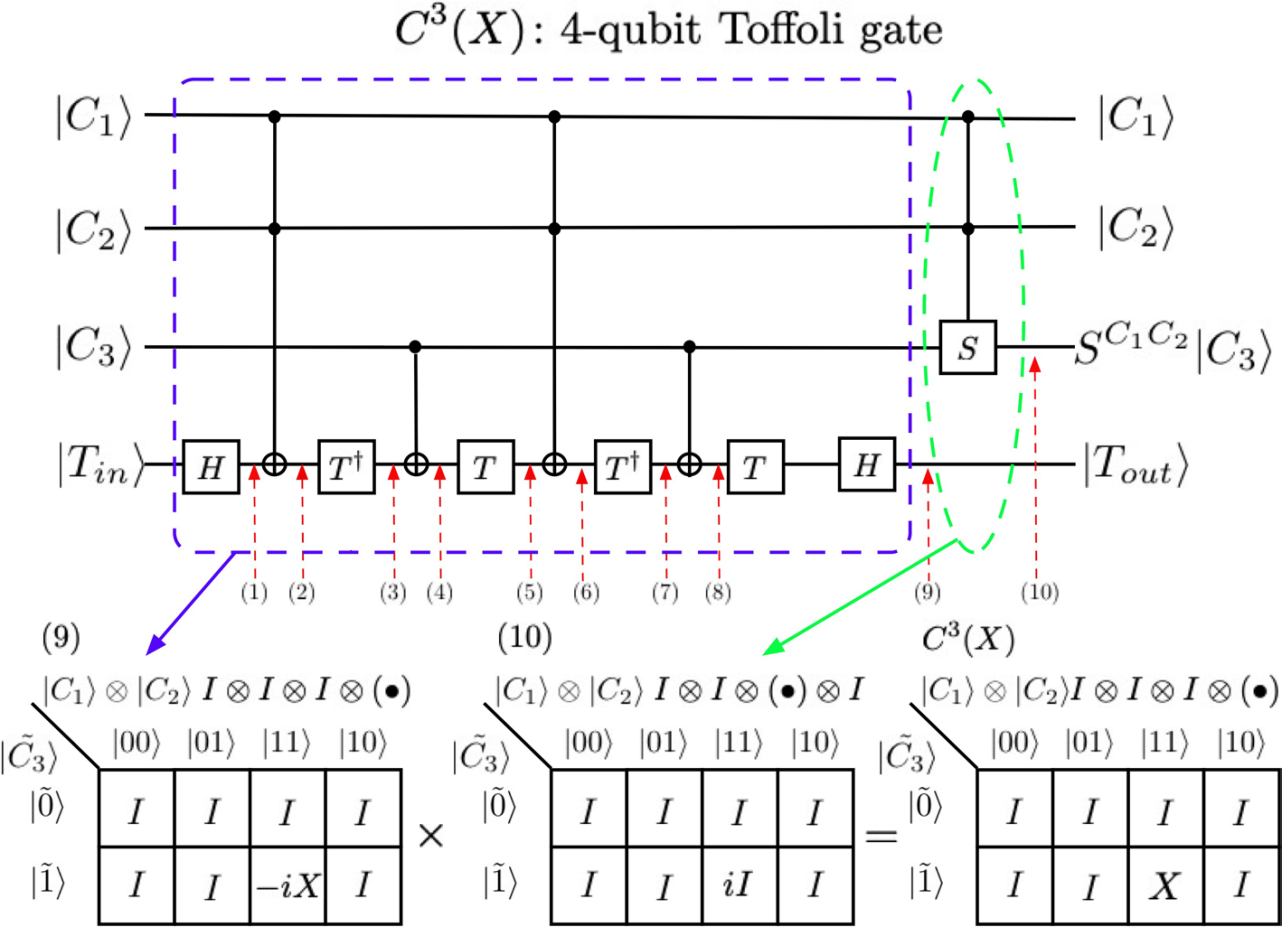
Reduced decomposition of $C^{3} (X)$. The reduced gate has 11 elements which are equivalent to 16 CNOT gates and 29 single qubit gates. This is almost 60% reduction of the number of elementary gates to implement of $C^{3} (X)$ from the original representation with 106 elementary gates. We also show three QKMs for this reduced representation. The first QKM is that of the partial circuits enclosed by blue-dashed line. The entries of the QKM are in the form of $I \otimes I \otimes I \otimes \left( \bullet \right)$ and obtained from the quantum states at point (9). The second QKM corresponds to the partial circuits enclosed by green-dashed line and the entries of the QKM are in the form of $I \otimes I \otimes \left( \cdot \right) \otimes I$ and obtained from the above analysis. The final QKM is that of the 4-qubit Toffoli gate $C^{3} (X)$ and obtained by the multiplication of the first two QKMs defined by equation (S19).

In Fig. 3, we show, by direct calculation, that the bottom circuits of Fig. 2 is indeed a four-qubit Toffoli gates in which $\left| {T_{out} } \right\rangle = X\left| {T_{in} } \right\rangle$ if and only if $C_{1} = C_{2} = C_{3} = 1$ otherwise the target bit $\left| {T_{in} } \right\rangle$ is not changed. In order to prove that we first calculate the quantum states at points (1), (2), …, (10) marked in the bottom of the circuits in Fig. 3. We describe the changes of the target qubit at each point as:

(1) $H\left| {T_{in} } \right\rangle$, (2) $X^{{C_{1} C_{2} }} H\left| {T_{in} } \right\rangle$, (3) $T^{\dag } X^{{C_{1} C_{2} }} H\left| {T_{in} } \right\rangle$, (4) $X^{{C_{3} }} T^{\dag } X^{{C_{1} C_{2} }} H\left| {T_{in} } \right\rangle$, (5) $TX^{{C_{3} }} T^{\dag } X^{{C_{1} C_{2} }} H\left| {T_{in} } \right\rangle$, (6) $X^{{C_{1} C_{2} }} TX^{{C_{3} }} T^{\dag } X^{{C_{1} C_{2} }} H\left| {T_{in} } \right\rangle$, (7) $T^{\dag } X^{{C_{1} C_{2} }} TX^{{C_{3} }} T^{\dag } X^{{C_{1} C_{2} }} H\left| {T_{in} } \right\rangle$, (8) $X^{{C_{3} }} T^{\dag } X^{{C_{1} C_{2} }} TX^{{C_{3} }} T^{\dag } X^{{C_{1} C_{2} }} H\left| {T_{in} } \right\rangle$, (9) $HTX^{{C_{3} }} T^{\dag } X^{{C_{1} C_{2} }} TX^{{C_{3} }} T^{\dag } X^{{C_{1} C_{2} }} H\left| {T_{in} } \right\rangle$, and (10) $S^{{C_{1} C_{2} }} \left| {C_{3} } \right\rangle$. Here $X^{\alpha } = X$ when $\alpha = 1$ and $X^{\alpha } = I$ when $\alpha = 0$. $S^{\alpha }$ is defined in the same way. Furthermore $X^{{C_{1} C_{2} }} = X$ for $C_{1} C_{2} = 1$ and $X^{{C_{1} C_{2} }} = I$ for $C_{1} C_{2} = 0$(SI). Let’s consider the possible combination of input qubits. When $C_{1} C_{2} = 0$ and $C_{3} = 0$, then $HTX^{{C_{3} }} T^{\dag } X^{{C_{1} C_{2} }} TX^{{C_{3} }} T^{\dag } X^{{C_{1} C_{2} }} H = HTT^{\dag } TT^{\dag } H = HH = I$; When $C_{1} C_{2} = 0$ and $C_{3} = 1$, then $HTX^{{C_{3} }} T^{\dag } X^{{C_{1} C_{2} }} TX^{{C_{3} }} T^{\dag } X^{{C_{1} C_{2} }} H = HTXT^{\dag } TXT^{\dag } H = I$; When $C_{1} C_{2} = 1$ and $C_{3} = 0$, then $HTX^{{C_{3} }} T^{\dag } X^{{C_{1} C_{2} }} TX^{{C_{3} }} T^{\dag } X^{{C_{1} C_{2} }} H = HTT^{\dag } XTT^{\dag } XH = I$; For $C_{1} C_{2} = 1$, $C_{3} = 1$, then $HTX^{{C_{3} }} T^{\dag } X^{{C_{1} C_{2} }} TX^{{C_{3} }} T^{\dag } X^{{C_{1} C_{2} }} H = HTXT^{\dag } XTXT^{\dag } XH = - iX$. On the other hand, when $C_{1} C_{2} = 1$ and $C_{3} = 0$, $S\left| 0 \right\rangle = \left| 0 \right\rangle$ and $S\left| 1 \right\rangle = i\left| 1 \right\rangle$. Therefore, $\left| 1 \right\rangle \otimes \left| 1 \right\rangle \otimes \left| 1 \right\rangle \otimes \left| {T_{in} } \right\rangle$ becomes $\left| 1 \right\rangle \otimes \left| 1 \right\rangle \otimes \left( {i\left| 1 \right\rangle } \right) \otimes \left( { - iX\left| {T_{in} } \right\rangle } \right) = \left| 1 \right\rangle \otimes \left| 1 \right\rangle \otimes \left| 1 \right\rangle \otimes \left( {X\left| {T_{in} } \right\rangle } \right)$, thus proving that the reduced quantum circuits is indeed four-qubit Toffoli gate.

In Fig. 3, we also show the three QKMs for this reduced representation from the analysis we provided above. The first QKM is that of the partial circuit enclosed by blue-dashed line. The entries of the QKM are in the form of $I \otimes I \otimes I \otimes \left( \cdot \right)$ and are obtained from the quantum states at point (9). The second QKM corresponds to the partial circuit enclosed by green-dashed line and the entries of the QKM are in the form of $I \otimes I \otimes \left( \bullet \right) \otimes I$ and obtained from the above analysis. The final QKM is that of the four-qubit Toffoli gate $C^{3} (X)$ and is obtained by the multiplication of the first two QKMs defined by equation (S9).

**Figure 4:**
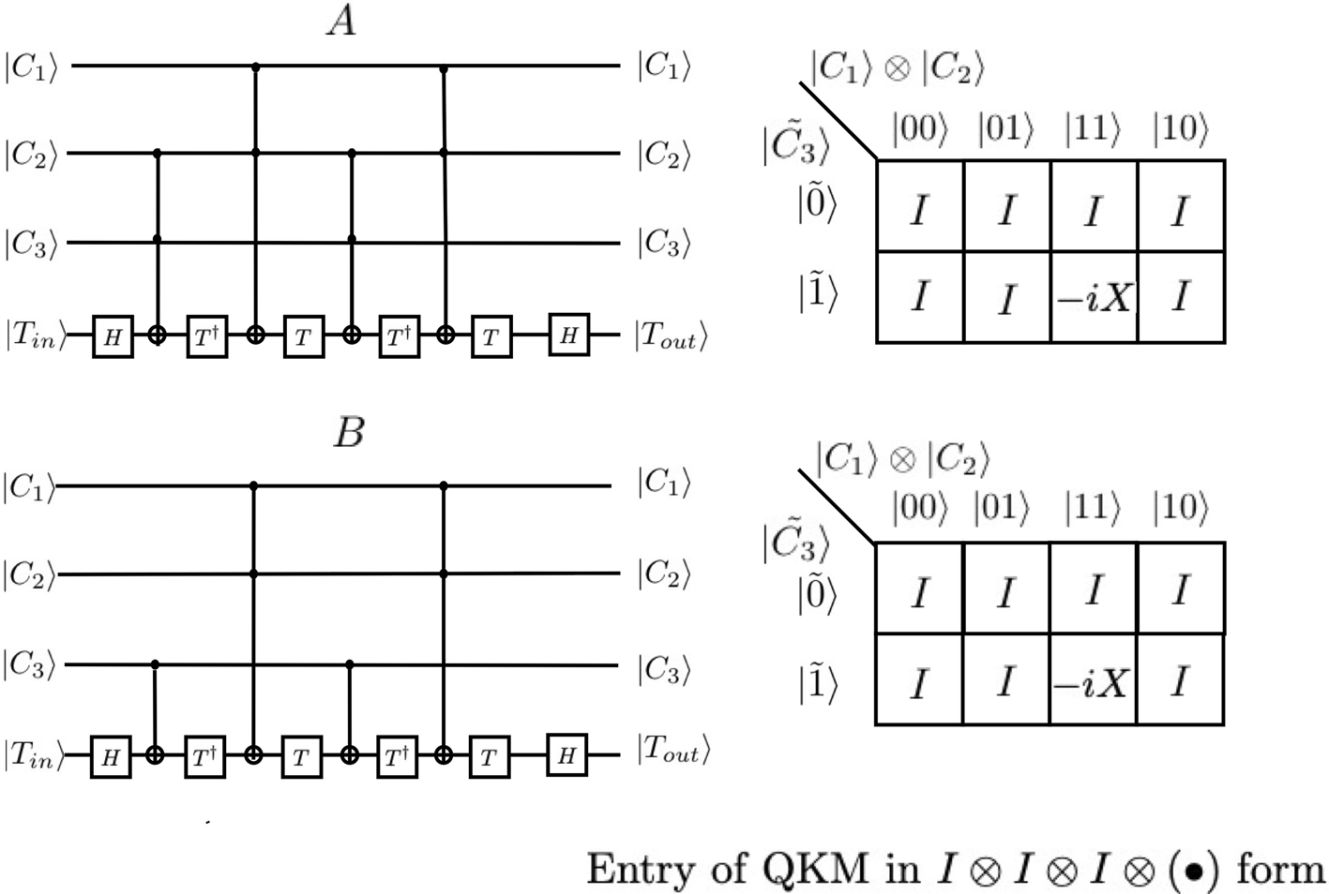
We show that the first two quantum circuits are equivalent to $C^{3} (X)$. In order to achieve this, we first show that partial circuits A (enclosed by red-dashed line) and B (enclosed by blue-dashed line) of Fig. 2 have the same QKM in Fig. 4. The entries of the QKM are in the form of $I \otimes I \otimes I \otimes \left( \cdot \right)$.

**Figure 5:**
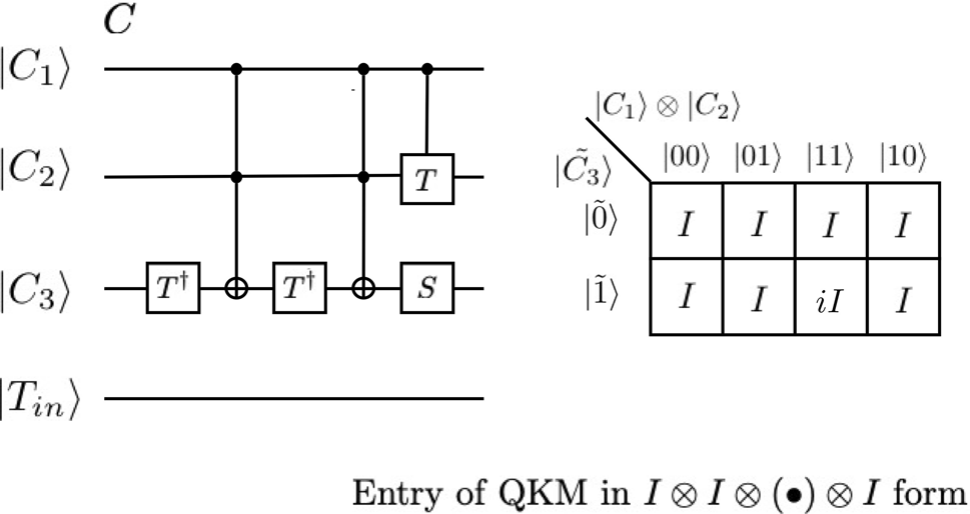
We show the QKM of a partial circuits C (enclosed by green-dashed line) of Fig. 2. The entries of the QKM are in the form of $I \otimes I \otimes \left( \cdot \right) \otimes I$. This QKM is also the same as that of a partial circuit D enclosed by orange-dashed line in Fig. 2.

In Figs. 4 and 5, we show that the first two quantum circuits shown in Fig. 2 are equivalent to $C^{3} (X)$, by the equivalency of their QKM representations In order to achieve this, we first show that partial circuits A (enclosed by red-dashed line) and B (enclosed by blue-dashed line) of Fig. 2 have the same QKM in Fig. 4. The entries of the QKM are in the form of $I \otimes I \otimes I \otimes \left( \cdot \right)$. In Fig. 5, we show the QKM of a partial circuits C (enclosed by green-dashed line) of Fig. 2. The entries of the QKM are in the form of $I \otimes I \otimes \left( \cdot \right) \otimes I$. This QKM is also the same as that of a partial circuit D enclosed by the orange-dashed line in Fig. 2. Detailed calculation of QKM entries is given in the SI.

The procedure for the reduction of the general $C^{m - 1} (X)$ gate is as follows:

1. Find the sub-circuit with the largest number of $C^{m - 2} (X)$ gates. $C^{m - 2} (X)$ are located altenatively with other unitary gate.
2. Replace half of $C^{m - 2} (X)$ gates by $C^{m - 3} (X)$ gates; check QKM equivalency.
3. Replace half of $C^{m - 3} (X)$ gates by $C^{m - 4} (X)$ gates; check QKM equivalency.
4. Continue the process until one reaches only $C^{1} (X)$ gates with equivalent QKM.
5. Repeat steps 1 to 4 until one cannot further reduce the circuit.

One needs to check the QKM when you reduce the circuit in every step. Figure S7 of SI shows the five-qubit Toffoli gate constructed with the minimum number of elemental gates using this technique. If we denote $\chi_{m} \left\{ {C^{m} (X)} \right\}$ the number of elemental gates needed to construct $C^{m} (X)$ gate, we obtain, $\chi_{2} \left\{ {C^{2} (X)} \right\} = 16$, $\chi_{3} \left\{ {C^{3} (X)} \right\} = 45$ and $\chi_{4} \left\{ {C^{4} (X)} \right\} = 115$ for our QKM based technique. Until now the simplest known decomposition^13,37^ of the five-qubit Toffoli gate requires 50 two-qubit gates or 250 elemental gates. Our decomposition of five-qubit Toffoli gate is 46% smaller than that of the simplest known decomposition.

**Figure 6:**
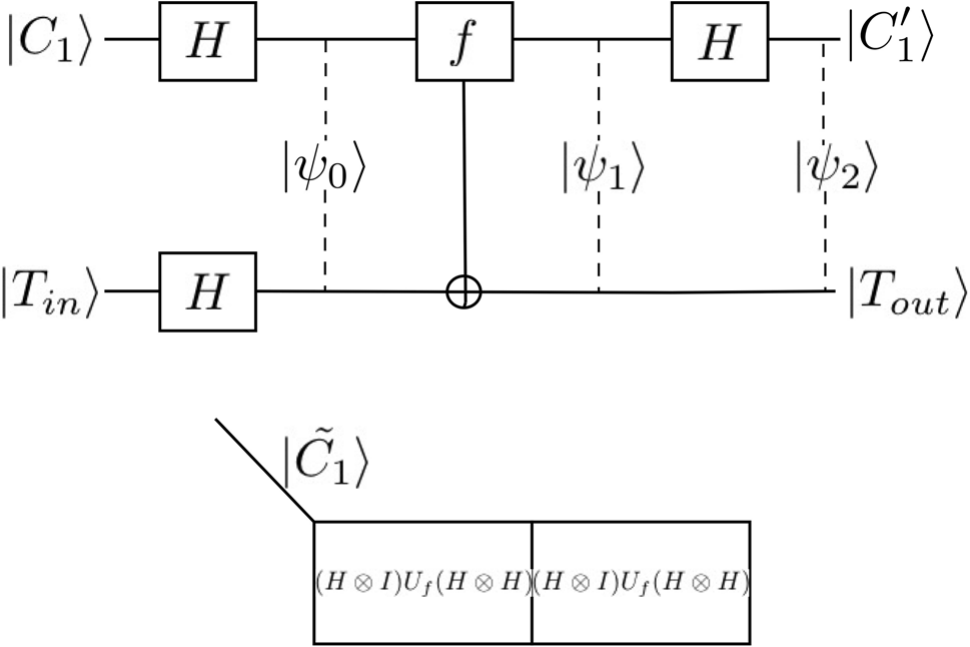
Implementation of the Deutsch algorithm and corresponding QKM.

It would be interesting to consider the potential application of QKM to quantum algorithms. Figure 6 shows the elementary implementation of the Deutsch algorithm^32^ and its corresponding QKM. Here $|\psi_{0} \rangle = \left( {H \otimes H} \right)\left( {\left| {C_{1} } \right\rangle \otimes \left| {T_{in} } \right\rangle } \right)$, $|\psi_{1} \rangle = U_{f} |\psi_{0} \rangle$, $|\psi_{2} \rangle = (H \otimes I)|\psi_{1} \rangle$ and $U_{f} (|x\rangle \otimes |y\rangle ) = |x\rangle \otimes |y \oplus f(x)\rangle$. For example if $|C_{1} \rangle = |0\rangle$, $|T_{in} = |1\rangle$, $f(0) = 0$ and $f(1) = 1$, then we obtain $|\psi_{2} \rangle = \frac{1}{\sqrt 2 }|1\rangle \otimes (|0\rangle - |1\rangle )$. We can expand this Deutsch algorithm to the five-qubit case. We first apply the Walsh-Harmard transformation to the register. Then we have the state $|\psi_{1} \rangle = (H \otimes H \otimes H \otimes H \otimes I)|\psi_{0} \rangle$. Then apply the $f(x)$-controlled NOT gate on the register which is a $U_{f}$ gate. If we choose this gate as a five-qubit Toffoli gate, then we have 46% reduction in the quantum circuit complexity which could being almost 200% speedup. By performing quantum circuits studies on IBM’s 20-qubit ‘Poughkeepsie’ architecture, one of the authors (P. M. A.) found that a single CNOT operation can be reliably performed in this NISQ environment^40^. The comparison of the QKM reduced circuits and the conventional circuits on the real NISQ machine such as IBM-Q will be the subject of future study.

## Discussion

The present results offer an efficient methodology for the design of complex quantum circuits which are the building blocks of quantum computers and quantum information processors. The lessons from the development of classical microelectronics taught us that the separation of device technology and the systems by means of an invariant interface to simplify the design is an essential and outstanding step to cope with the complexity of the system. Our hypothesis is that this principle of hierarchical design will be, in some form, valid for quantum architecture. This can begin to be impactful especially with the advent of prototype quantum computers with around 50 available qubits from IBM, Google and Intel, to name a few, where qubits are the most expensive resources. Following this motivation, we demonstrated here a ~ 60% reduction in the number of elementary gates to implement four-qubit quantum gate using the QKM. The first step of the reduction of four-qubit Toffoli gate $C^{3} (X)$ can be achieved by replacing half of the three-qubit Toffoli gates $C^{2} (X)$ embedded in a 4-qubit quantum circuit by two-qubit CNOT gates $C^{1} (X)$, as can be seen by the Fig. 4. Further simplification can be achieved by replacing the phase shift circuit C by a 2-qubit S gate embedded in a 4-qubit quantum circuit as can be seen Fig. 2. We also demonstrated the decomposition of five-qubit Toffoli gate with 46% reduction in the number of elementary gate when compared with known simplest decomposition. We hope that the introduction of the QKM proposed in this work would lead to further development of quantum information science and engineering by separating the quantum circuit design and the device technology. The use of the QKM may help accelerate the solid-state implementation of quantum computers because the proposed scheme utilizes most of the conventional design methodology.

## Method

### Compact qubit notation

We start by recalling a controlled unitary gate $C^{1} (U)$ defined in the $\left\{ {\left| {00} \right\rangle ,\;\left| {01} \right\rangle ,\;\left| {10} \right\rangle ,\;\left| {11} \right\rangle } \right\}$ basis^1^

6 $$C^{1} (U) = \left( {\begin{array}{*{20}l} 1 & 0 & 0 & 0 \\ 0 & 1 & 0 & 0 \\ 0 & 0 & {u_{00} } & {u_{01} } \\ 0 & 0 & {u_{10} } & {u_{11} } \\ \end{array} } \right)$$

where $$U = \left( {\begin{array}{*{20}l} {u_{00} } & {u_{01} } \\ {u{}_{10}} & {u_{11} } \\ \end{array} } \right)$$.

The controlled unitary gate satisfies the following switching function properties:

7 $$\begin{gathered} C^{1} (U)\left| {00} \right\rangle = \left| {00} \right\rangle , \hfill \\ C^{1} (U)\left| {01} \right\rangle = \left| {01} \right\rangle , \hfill \\ C^{1} (U)\left| {10} \right\rangle = u_{00} \left| {10} \right\rangle + u_{10} \left| {11} \right\rangle , \hfill \\ C^{1} (U)\left| {11} \right\rangle = u_{01} \left| {10} \right\rangle + u_{11} \left| {11} \right\rangle . \hfill \\ \end{gathered}$$

It is well known that we may expand any operators by outer product of the complete basis, i.e., $C^{1} (U) = \sum\nolimits_{n} {C^{1} (U)\left| n \right\rangle \left\langle n \right|}$ with $\sum\nolimits_{n} {\left| n \right\rangle \left\langle n \right|} = I_{n}$ where $I_{n}$ is the $n \times n$ identity matrix in an *n*-dimensional Hilbert space and $\left| n \right\rangle \left\langle n \right| \equiv \left| n \right\rangle \otimes \left\langle n \right|$ with $\otimes$ denoting a tensor product. Equation (7) can be rewritten as

8 $$C^{1} (U)\left| {xy} \right\rangle = \left\{ {\begin{array}{*{20}l} {\left| {xy} \right\rangle } & {\quad {\text{ for }}x = 0,} \\ {u_{0y} \left| {x0} \right\rangle + u_{1y} \left| {x1} \right\rangle { = }\sum\limits_{{k = \{ 0,1\} }} {\left| {x\,k} \right\rangle } \,u_{ky} } & {\quad {\text{ for }}x = 1.} \\ \end{array} } \right.$$

We found that instead of counting the cases for the $\left\{ {\left| {00} \right\rangle ,\;\left| {01} \right\rangle ,\;\left| {10} \right\rangle ,\;\left| {11} \right\rangle } \right\}$ basis, we obtain the same results by employing a compact 2-qubit basis $\left\{ {\left| {\tilde{0}} \right\rangle_{2} ,\left| {\tilde{1}} \right\rangle_{2} } \right\}$ which are defined by.

9 $$\left| {\tilde{0}} \right\rangle_{2} \equiv \left| 0 \right\rangle \otimes I = \left( {\begin{array}{*{20}l} 1 & {\quad 0} \\ 0 & {\quad 1} \\ 0 & {\quad 0} \\ 0 & {\quad 0} \\ \end{array} } \right) = \left( {\begin{array}{*{20}l} I \\ O \\ \end{array} } \right)\quad {\text{and}}\quad \left| {\tilde{1}} \right\rangle_{2} \equiv \left| 1 \right\rangle \otimes I = \left( {\begin{array}{*{20}l} 0 & {\quad 0} \\ 0 & {\quad 0} \\ 1 & {\quad 0} \\ 0 & {\quad 1} \\ \end{array} } \right) = \left( {\begin{array}{*{20}l} O \\ I \\ \end{array} } \right).$$

Here, for simplicity, we denote *I* and *O* for the identity and null $2 \times 2$ matrices in 2-dimensional Hilbert space; respectively. In this compact 2-qubit notation, the first and the second column of $\left| {\tilde{0}} \right\rangle_{2}$ corresponds to two qubit states $\left| {00} \right\rangle$ and $\left| {01} \right\rangle$; respectively. Likewise the first and the second column of $\left| {\tilde{1}} \right\rangle_{2}$ corresponds to the two qubit states $\left| {10} \right\rangle$ and $\left| {11} \right\rangle$; respectively. The compact 2-qubit is a short-hand notation representing two qubits with common first qubit index such as $0$ in $\left| {00} \right\rangle$ and $\left| {01} \right\rangle$, denoted as $\left| {\tilde{0}} \right\rangle_{2}$. The compact 2-qubits satisfies the following closure relation:

10 $$\left| {\tilde{0}} \right\rangle_{2} \left\langle {\tilde{0}} \right| + \left| {\tilde{1}} \right\rangle_{2} \left\langle {\tilde{1}} \right| = I_{4} ,$$

where

11 $$\left| {\tilde{0}} \right\rangle_{2} \left\langle {\tilde{0}} \right| = \left| {\tilde{0}} \right\rangle_{2} \otimes {}_{2}\left\langle {\tilde{0}} \right| = \left( {\begin{array}{*{20}l} I \\ O \\ \end{array} } \right)\left( {\begin{array}{*{20}l} I & O \\ \end{array} } \right) = \left( {\begin{array}{*{20}l} I & {\quad O} \\ O & {\quad O} \\ \end{array} } \right) = \left( {\begin{array}{*{20}l} 1 & {\quad 0} & {\quad 0} & {\quad 0} \\ 0 & {\quad 1} & {\quad 0} & {\quad 0} \\ 0 & {\quad 0} & {\quad 0} & {\quad 0} \\ 0 & {\quad 0} & {\quad 0} & {\quad 0} \\ \end{array} } \right),$$

and

12 $$\left| {\tilde{1}} \right\rangle_{2} \left\langle {\tilde{1}} \right| = \left| {\tilde{1}} \right\rangle_{2} \otimes {}_{2}\left\langle {\tilde{1}} \right| = \left( {\begin{array}{*{20}l} O \\ I \\ \end{array} } \right)\left( {\begin{array}{*{20}l} O & I \\ \end{array} } \right) = \left( {\begin{array}{*{20}l} O & {\quad O} \\ O & {\quad I} \\ \end{array} } \right) = \left( {\begin{array}{*{20}l} 0 & {\quad 0} & {\quad 0} & {\quad 0} \\ 0 & {\quad 0} & {\quad 0} & {\quad 0} \\ 0 & {\quad 0} & {\quad 1} & {\quad 0} \\ 0 & {\quad 0} & {\quad 0} & {\quad 1} \\ \end{array} } \right).$$

Here $I_{4}$ is the identity matrix in a four-dimensional Hilbert space.

For a three-qubit gate, the compact 3-qubits are defined as

13 $$\left| {0\tilde{0}} \right\rangle_{3} \equiv \left| {00} \right\rangle \otimes I,\;\;\left| {0\tilde{1}} \right\rangle_{3} \equiv \left| {01} \right\rangle \otimes I,\;\; \, \left| {1\tilde{0}} \right\rangle_{3} \equiv \left| {10} \right\rangle \otimes I,\;\; \, \left| {1\tilde{1}} \right\rangle_{3} \equiv \left| {11} \right\rangle \otimes I.$$

For example,

14 $$\left| {0\tilde{0}} \right\rangle_{3} = \left| {00} \right\rangle \otimes I = \left| 0 \right\rangle \otimes \left| {\tilde{0}} \right\rangle_{2} = \left( {\begin{array}{*{20}l} I \\ O \\ O \\ O \\ \end{array} } \right) = \left( {\left| {000} \right\rangle ,\;\;\left| {001} \right\rangle } \right),$$

where the first and the second column corresponds to 3-qubit states $\left| {000} \right\rangle$ and $\left| {001} \right\rangle$; respectively. It is straightforward to show that the compact 3-qubits satisfy the following completeness relation.

15 $$\left| {0\tilde{0}} \right\rangle_{3} \left\langle {0\tilde{0}} \right| + \left| {1\tilde{0}} \right\rangle_{3} \left\langle {1\tilde{0}} \right| + \left| {0\tilde{1}} \right\rangle_{3} \left\langle {0\tilde{1}} \right| + \left| {1\tilde{1}} \right\rangle_{3} \left\langle {1\tilde{1}} \right| = I_{8} .$$

## Supplementary information

Supplementary Information.

## Data Availability

All data generated or analyzed during this study are included in this article (and its supplementary information files).

## References

[1] Nakamura Y, Pashkin YA, Tsai JS (1999). Coherent control of macroscopic quantum states in a single-Cooper-pair box. Nature.

[2] Vion D, Aassime A, Cottet A, Joyez P, Pothier H, Urbina C, Esteve D, Dovoret MH (2002). Manipulating the quantum state of an electrical circuits. Science.

[3] Yamamoto T, Astafiev O, Nakamura Y, Averin DV, Tsai JS (2003). Quantum oscillations in two coupled charge qubits. Nature.

[4] Yamamoto T, Pashkin YA, Astafiev O, Nakamura Y, Tsai JS (2003). Demonstration of conditional gate operation using superconducting charge qubits. Nature.

[5] Kane BE (1998). A silicon-based nuclear spin quantum computer. Nature.

[6] Loss D, DiVincenzo DP (1998). Quantum computation with quantum dots. Phys. Rev. A.

[7] Burkard G, Loss D, DiVincenzo DP (1999). Coupled quantum dots as quantum gates. Phys. Rev. B.

[8] DiVincenzo DP, Bacon D, Kempe J, Burkard G, Whaley KB (2000). Universal quantum computation with the exchange interaction. Nature.

[9] Fujisawa T, Austing DG, Tokura DY, Hirayama Y, Tarucha S (2000). Allowed and forbidden transitions in artificial hydrogen and helium atoms. Nature.

[10] Koppens FHL, Buizert C, Tielrooij KJ, Vink IT, Nowack KC, Meunier T, Kouwenhoven LPL, Vandersypen LMK (2006). Driven coherent oscillations of a single electron spin in a quantum dot. Nature.

[11] Ahn D (2005). Intervalley interactions in Si Quantum dots. J. Appl. Phys..

[12] Barenco A, Bennett CH, Cleve R, DiVincenzo DP, Margolus N, Shor P, Sleator T, Smolin JA, Weinfurter H (1995). Elementary gates for quantum computation. Phys. Rev. A.

[13] Lanyon BP (2009). Simplifying quantum logic using higher-dimensional Hilbert spaces. Nat. Phys..

[14] Preskill J (2018). Quantum computing in the NISQ era and beyond. Quantum.

[15] Wang Y, Li Y, Yin Z-Q, Zeng B (2018). 16-qubit IBM universal quantum computer can be fully entangled. Npj Quantum Inf..

[16] Bravyi S, Gosset D, Konig R (2018). Quantum advantage with shallow circuits. Science.

[17] Arute F (2019). Quantum supremacy using a programmable superconducting processor. Nature.

[18] Gyongyosi L (2020). Quantum state optimization and computational pathway evaluation for gate-model quantum computers. Sci. Rep..

[19] Gyongyosi L, Imre S (2020). Circuit depth reduction for gate-model quantum computers. Sci. Rep..

[20] Gyongyosi L, Imre S (2020). Optimizing high-efficiency quantum memory with quantum machine learning for near term-quantum devices. Sci. Rep..

[21] Gyongyosi L, Imre S (2019). Quantum circuit design for objective function maximization in gate-model quantum computers. Quantum Inf. Process..

[22] Gyongyosi L, Imre S (2019). A survey on quantum computing technology. Comput. Sci. Rev..

[23] Gyongyosi L, Imre S (2019). Dense quantum measurement theory. Sci. Rep..

[24] Gyongyosi L, Imre S, Nguyen HV (2018). A survey on quantum channel capacities. IEEE Commun. Surv. Tutor..

[25] Farhi, E., Goldstone, J., Gutmann, S. & Zhou, L. The quantum approximate optimization algorithm and the Sherrington–Kirkpatrick model at infinite size. Preprint at https://arXiv.org/abs/1910.08187 (2019).

[26] Farhi, E., Gamarnik, D. & Gutmann, S. The quantum approximate optimization algorithm need to see the whole graph. Preprint at https://arXiv.org/abs/2004.09002 (2020).

[27] Farhi, E. & Neven, H. Classification with quantum neural networks on near term processors. Preprint at https://arXiv.org/abs/1802.06002 (2018).

[28] Farhi, E., Goldstone, J., Gutmann, S. & Neven, H. Quantum algorithms for fixed qubit architectures. Preprint at https://arXiv.org/abs/1803.06199 (2018).

[29] Farhi, E., Goldstone, J. & Gutmann, S. A quantum approximate optimization algorithm. Preprint at https://arXiv.org/abs/1411.4028 (2014).

[30] Lloyd, S. Quantum approximate optimization is computationally universal. Preprint at https://arXiv.org/abs/1812.11075 (2018).

[31] Mead C, Conway L (1980). Physics of Computational Systems, an Introduction to VLSI Systems.

[32] Mano MM (1979). Digital Logic and Computer Design.

[33] Wang, S. A., Lu, C. Y., Tsai, I. M. & Kuo, S. Y. Modified Karnaugh map for quantum Boolean circuits construction. In *Proceedings of the 3rd IEEE Conference on Nanotechnology*, Vol. 2, 651–654 (2003)

[34] Ahn, D. Quantum Karnaugh map.US Patent 8,671,369 (2014).

[35] Roth CH, Kinney LL (2010). Fundamentals of Logic Design.

[36] Nakahara M, Ohmi T (2008). Quantum Computing.

[37] Nielsen MA, Chuang IL (2000). Quantum Computation and Quantum Information.

[38] Krauss B, Cirac JI (2001). Optimal creation of entanglement using a two-qubit gate. Phys. Rev. A.

[39] DiVincenzo DP (1995). Two-bit gates are universal for quantum computation. Phys. Rev. A.

[40] Koch, D., Martin, B., Patel, S., Wessing, L. & Alsing, P. M. Benchmarking qubit quality and critical subroutines on IBM’s 20 qubit device. Preprint at https://arXiv.org/abs/2003.01009 (2020).

