# Supplementary material for: Quantum circuit optimization using quantum Karnaugh map
Source: Sci Rep. 2020 Sep 24;10:15651. doi: 10.1038/s41598-020-72469-7 (PMC7518448; doi:10.1038/s41598-020-72469-7)
Supplement: Supplementary file 1 — Supplementary Information. [file 41598_2020_72469_MOESM1_ESM.docx]

**Supplementary Information for**

**Quantum circuit optimization using quantum Karnaugh map**

J.-H. Bae^1^, Paul M. Alsing^2^, Doyeol Ahn^1,3,4*^ and Warner A. Miller^3^

^1^Department of Electrical and Computer Engineering,

University of Seoul, 163 Seoulsiripdae-ro, Tongdaimoon-Gu, Seoul 02504, Korea

^2^Air Force Research Laboratory, Information Directorate, Rome NY 13441, USA

^3^Department of Physics, Florida Atlantic University, Boca Raton, FL 33431, USA

^4^Peta Lux Inc, 12 Yanghyeon-ro 405 beon-gill, Seongnam 13438, Gyeonggi-do, Korea

*To whom correspondence should be addressed.

E-mail: [dahn@uos.ac.kr](mailto:dahn@uos.ac.kr) ; [daveahn@ymail.com](mailto:daveahn@ymail.com)

**1. Elementary operations on the compact qubits and quantum Karnaugh map**

From $|\tilde{0}\rangle_{2}\langle\tilde{0}|+|\tilde{1}\rangle_{2}\langle\tilde{1}|=I_{4}$, one can expand $C^{1}(U)$in compact 2-qubit basis as follows

$C^{1}(U)=C^{1}(U)|\tilde{0}\rangle_{2}\langle\tilde{0}|+C^{1}(U)|\tilde{1}\rangle_{2}\langle\tilde{1}|=\left(\begin{matrix} I & O \\ O & U \end{matrix}\right)$, (S1)

where $U=\left(\begin{matrix} u_{00} & u_{01} \\ u{}_{10} & u_{11} \end{matrix}\right)$. One can find that equation (S11) is identical to the definition of $C^{1}(U)$ given by equation (6).

The action of a controlled unitary operator $C^{1}(U)$ on the compact qubits is then given by

$C^{1}(U)|\tilde{0}\rangle_{2}=\left(\begin{matrix} I & O \\ O & U \end{matrix}\right)\left(\begin{matrix} I \\ O \end{matrix}\right)=\left(\begin{matrix} I \\ O \end{matrix}\right)=\left(\begin{matrix} 1 & 0 \\ 0 & 1 \\ 0 & 0 \\ 0 & 0 \end{matrix}\right)=\left(I\otimes I\right)|\tilde{0}\rangle_{2}$,

and $C^{1}(U)|\tilde{1}\rangle_{2}=\left(\begin{matrix} I & O \\ O & U \end{matrix}\right)\left(\begin{matrix} O \\ I \end{matrix}\right)=\left(\begin{matrix} O \\ U \end{matrix}\right)=\left(\begin{matrix} 0 & 0 \\ 0 & 0 \\ u_{00} & u_{01} \\ u_{10} & u_{11} \end{matrix}\right)=\left(I\otimes U\right)|\tilde{1}\rangle_{2}$, (S2)

If we compare equations (7) and (S2), we can see that the same results can be expressed with the number of equations reduced by the factor of 2. The tabular representation of the above results for each input is denoted as quantum Karnaugh map^2^.

Fig. S1 shows the quantum Karnaugh map (QKM) of the quantum circuit for a two- qubit controlled unitary $C^{1}(U)$gate based on equation (S2). The upper part of this figure describes the action of the control qubit $|C\rangle$on the controlled unitary circuit acting on the input qubit $|T_{in}\rangle$ that produces the output state $|T_{out}\rangle$as described by equation (7). The lower part of the figure shows the quantum Karnaugh map (QKM). In this QKM, the control qubit and the input qubit is combined as a compact control qubit $|\tilde{C}\rangle$and the output of the gate is described in the box as $I\otimes I$and that corresponds to the input states, and ; respectively, as described by equation (S2).

Fig. S2 shows the 3-qubit Toffoli gate denoted as $C^{2}(X)$ in which the target qubit is transformed as $X^{C_{1}C_{2}}|T_{in}\rangle$ where $X^{C_{1}C_{2}}=X$ for $C_{1}C_{2}=1$ and $X^{C_{1}C_{2}}=I$ for $C_{1}C_{2}=0$. The 3-qubit Toffoli gate is expanded in the compact 3-qubit basis as

(S3)

In Fig. S2, we show the corresponding QKM corresponding to $C^{2}(X)$ in the lower part of the figure. In this QKM, the compact qubit combines both $|C_{2}\rangle$ and $|T_{in}\rangle$ as a tensor product.

In Fig. S3, we illustrate the Controlled-NOT gates or $C^{1}(X)$ gates as embedded in the 3-qubit quantum circuit along with their corresponding QKMs. The left side of the figure corresponds to case of $C^{1}(X)$ gates with a control qubit $|C_{1}\rangle$ without operations on $|C_{2}\rangle$. On the other hand, the right side of the case of $C^{1}(X)$ gates with a control qubit $|C_{2}\rangle$ without operations on $|C_{1}\rangle$.

Fig. S4 depicts a quantum circuit $G(U)$ that executes a unitary operation $U$ when one of the control qubits is in the state$|1\rangle$, i.e. when the value of the Exclusive-OR operation of the two control bits is 1 ($C_{1}\oplus C_{2}=1$). The corresponding QKM that is constructed from $G(U)$ is illustrated in the lower part of Fig. S4.

In this Fig. S4, $|C_{1}\rangle,|C_{2}\rangle$are the control qubits, $|T_{in}\rangle$ is the the input state (target qubit) and $|T_{out}\rangle$is the output qubit state. The operation of $G(U)$on 3 qubit states $\left\{|000\rangle,|001\rangle,|010\rangle,|011\rangle,|100\rangle,|101\rangle,|110\rangle,|111\rangle\right\}$ is given by

$\begin{matrix} G(U)|000\rangle=|000\rangle,G(U)|001\rangle=|001\rangle, \\ G(U)|010\rangle=u_{00}|010\rangle+u_{10}|011\rangle=I\otimes I\otimes U|010\rangle, \\ G(U)|011\rangle=u_{01}|010\rangle+u_{11}|011\rangle=I\otimes I\otimes U|011\rangle, \\ G(U)|100\rangle=u_{00}|100\rangle+u_{10}|101\rangle=I\otimes I\otimes U|100\rangle, \\ G(U)|101\rangle=u_{01}|100\rangle+u_{11}|101\rangle=I\otimes I\otimes U|101\rangle, \\ G(U)|110\rangle=|110\rangle,G(U)|111\rangle=|111\rangle. \end{matrix}$ (S4)

From equation (S4), the mathematical expression of the quantum circuit $G(U)$ in compact 3-qubit can be rewritten compactly as

$\begin{matrix} G(U)=\left(I\otimes I\otimes I\right)|0\tilde{0}\rangle\langle0\tilde{0}|+\left(I\otimes I\otimes U\right)|0\tilde{1}\rangle\langle0\tilde{1}| \\ \text{ }+\left(I\otimes I\otimes U\right)|1\tilde{0}\rangle\langle1\tilde{0}|+\left(I\otimes I\otimes I\right)|1\tilde{1}\rangle\langle1\tilde{1}|. \end{matrix}$ (S5)

In Fig. S4, the tensor product state of second control qubit $|C_{2}\rangle$and the $|T_{in}\rangle$is represented by the compact 2-qubit state $|\tilde{C}\rangle_{2}$. One can regard QKM as a simplified map to track the control qubits, input qubits and output qubits.

Expressing quantum circuits by QKM in the compact qubit representation simplifies the analysis of the composite circuits considerably.

For example, if we have two controlled unitary gates $G(U)$ and $H(U)$ given by

(S6)

and , (S7)

then the successive operation of $H(U)$ $G(U)$ followed by on the input qubits is given by

(S8)

Here the gate (Hadamard or Schur, element-wise) product $G\circ H$ is defined by

$\left[G\circ H\right]_{ij}≐\left[G\right]_{ij}\left[H\right]_{ij}$ (S9)

for gates with same number of rows and columns.

We illustrate in Fig. S5 the representation of a 3-qubit quantum circuit with the combinational rule defined by equations (S8) and (S9) and the resulting QKMs. This figure also shows the most important feature of the QKM. When we have a circuit consisting of the composition of $G(U)$and $H(U)$with the corresponding QKMs having same number of rows and columns, the final QKM is constructed from the element-wise product of equation (S19), where $\left[X\right]_{ij}$is the entry of the *i* th row and *j* th column of QKM corresponding to quantum circuit $X$. In Fig. S5, the partial circuit $H(U)$, acting on the input states in the far left side of the figure is enclosed with red dotted line and the partial circuit $G(U)$is acting on the output states of $H(U)$ are enclosed with blue dotted lines. Mathematically it is denoted as $G(U)$ acting on$H(U)$. In other words, for the quantum circuits the operation is from left to right, and for the mathematical expression the operation is the other direction from right to left with the input states on the right side of the mathematical expression.

Fig. S5 shows the quantum circuit manifesting $G\circ H$ as described in equations (S8) and (S9) where

$\begin{matrix} H(U)=\left(I\otimes I\otimes I\right)|0\tilde{0}\rangle\langle0\tilde{0}|+\left(I\otimes I\otimes I\right)|0\tilde{1}\rangle\langle0\tilde{1}| \\ \text{ }+\left(I\otimes I\otimes U\right)|1\tilde{0}\rangle\langle1\tilde{0}|+\left(I\otimes I\otimes U\right)|1\tilde{1}\rangle\langle1\tilde{1}|. \end{matrix}$ (S10)

From equations (S5), (S8), (S10) and we obtain

$\begin{matrix} \left(G\circ H\right)(U)=\left(I\otimes I\otimes I\right)|0\tilde{0}\rangle\langle0\tilde{0}|+\left(I\otimes I\otimes U\right)|0\tilde{1}\rangle\langle0\tilde{1}| \\ \text{ }+\left(I\otimes I\otimes U^{2}\right)|1\tilde{0}\rangle\langle1\tilde{0}|+\left(I\otimes I\otimes U\right)|1\tilde{1}\rangle\langle1\tilde{1}|, \end{matrix}$ (S11)

which is summarized in the final QKM of Fig. S5. The partial circuits *G(U)* (enclosed by red dashed line) corresponds to the CNOT gate which arises from the XOR operation on the second qubit. On the other hand, the partial circuits *H(U)* (enclosed by blue dashed line) corresponds to the AND operation between the first- and the second- qubit, which yields controlled unitary gate.

The operator for any controlled unitary gate $C^{m}(U)$for the *(m+1)*-qubits is given by^1^

$C^{m}(U)=\left(\begin{matrix} 1 & & & & & \\ & 1 & & & & \\ & & \ddots& & & \\ & & & 1 & & \\ & & & & u_{00} & u_{01} \\ & & & & u_{10} & u_{11} \end{matrix}\right)$, (S12)

and the operation of $C^{m}(U)$on the state $|x_{1}x_{2}\cdots x_{m}y\rangle$ is defined by

$\begin{matrix} C^{m}(U)|x_{1}x_{2}\cdots x_{m}y\rangle\\ =\left\{\begin{matrix} u_{0y}|x_{1}x_{2}\cdots x_{m}0\rangle+u_{1y}|x_{1}x_{2}\cdots x_{m}1\rangle\text{ if }\wedge_{k=1}^{m}x_{k}=1, \\ |x_{1}x_{2}\cdots x_{m}y\rangle\text{ if }\wedge_{k=1}^{m}x_{k}=0\text{ } \end{matrix}\right. \end{matrix}$ (S13)

for all $x_{1},x_{2},\cdots x_{m},y\in\left\{0,1\right\}$[13]. Here $\wedge_{k=1}^{m}x_{k}$ denotes the AND of Boolean variables $\left\{x_{k}\right\}$.

As in the case of the previous section the QKM can be constructed with compact *m*-qubit $|x_{1}x_{2}\cdots x_{m-1}\tilde{y}\rangle\equiv|x_{1}x_{2}\cdots x_{m-1}y\rangle\otimes I$ which will reduce the size of map by$2^{(m+1)}\times2^{(m+1)}/\left(2^{m}\times2^{m}\right)=4$. It is well known that any unitary operator $U$for a single qubit can be represented by $U=GP(\alpha)AXBXC$where A, B, C are arbitrary unitary operator for single qubit such that $A,B,C\in SU(2)$ with$ABC=I$, $X=\left(\begin{matrix} 0 & 1 \\ 1 & 0 \end{matrix}\right)$ and $GP(\alpha)=\left(\begin{matrix} 1 & 0 \\ 0 & e^{i\alpha} \end{matrix}\right)$where $\alpha$is the global phase.^1^ Here $X$ is the NOT operation and $GP(\alpha)$ is the general phase gate. In equation (S12), when $U=X$, we call this gate as $C^{m}(X)$ gate. In this paper, we use the notion of a gate and an operator, interchangeably. Once we decompose $C^{m}(U)$gate using QKM and determine the unitary operators $A,B,C$and the global phase factor$\alpha$, then an arbitrary unitary gate $C^{m}(U)$can be constructed. In the following, we would like to introduce the basic gates which will be used to decompose $C^{m}(U)$ or $C^{m}(X)$ for the representation of QKM:

$H=\frac{1}{\sqrt{2}}\left(\begin{matrix} 1 & 1 \\ 1 & -1 \end{matrix}\right),\text{ }S=\left(\begin{matrix} 1 & 0 \\ 0 & i \end{matrix}\right),\text{ }T=\left(\begin{matrix} 1 & 0 \\ 0 & e^{i\pi/4} \end{matrix}\right),\text{ }T^{\text{†}}=\left(\begin{matrix} 1 & 0 \\ 0 & e^{-i\pi/4} \end{matrix}\right)$, (S14)

Here $H$is the Hadamard gate, $S$ the phase gate and $T$is the $\pi/8$gate.^3^ These are the elementary single qubit gates acting on the two-qubit state. It was shown that these unitary operations on one qubit and the XOR gate (or CNOT gate) is sufficient for general quantum programming.^3-6^ For example, a phase shift gate defined previously which includes *S* and *T* gates is decomposed as^3^

$\begin{matrix} GP(\alpha)=e^{i\frac{\alpha}{2}}R_{z}\left(\alpha/2\right)X R_{z}\left(-\alpha/4\right)X R_{z}\left(-\alpha/4\right) \\ =e^{i\frac{\alpha}{2}}\left(\begin{matrix} e^{-i\alpha/2} & 0 \\ 0 & e^{i\alpha/2} \end{matrix}\right)\left(\begin{matrix} 0 & 1 \\ 1 & 0 \end{matrix}\right)\left(\begin{matrix} e^{i\alpha/4} & 0 \\ 0 & e^{-i\alpha/4} \end{matrix}\right)\left(\begin{matrix} 0 & 1 \\ 1 & 0 \end{matrix}\right)\left(\begin{matrix} e^{i\alpha/4} & 0 \\ 0 & e^{-i\alpha/4} \end{matrix}\right), \\ =\left(\begin{matrix} 1 & 0 \\ 0 & e^{i\alpha} \end{matrix}\right), \end{matrix}$ (S15)

and

$$I=ABC=R_{z}\left(\alpha/2\right)R_{z}\left(-\alpha/4\right)R_{z}\left(-\alpha/4\right)=\left(\begin{matrix} e^{-i\alpha/2} & 0 \\ 0 & e^{i\alpha/2} \end{matrix}\right)\left(\begin{matrix} e^{i\alpha/4} & 0 \\ 0 & e^{-i\alpha/4} \end{matrix}\right)\left(\begin{matrix} e^{i\alpha/4} & 0 \\ 0 & e^{-i\alpha/4} \end{matrix}\right),$$

for $R_{z}(\theta)=e^{-i \theta\sigma_{z}}=\left(\begin{matrix} e^{-i\theta} & 0 \\ 0 & e^{i\theta} \end{matrix}\right).$

Fig. S6 shows the elementary decomposition of $C^{2}(X)$gate so called 3-qubit Toffoli gate and its its corresponding QKM representation. The elementary decomposition of $C^{2}(X)$is described in the Fig. 4.9 of Nielsen and Chang.^3^ From equations (S9), (S13) and (S14), the input-output relation of the partial circuits $G^{(1)}$ (enclosed by red dashed-line) in Fig. S6 can be expressed as

, (S16)

where

(S17)

Here $X^{\tilde{C}_{2}}$is a two qubit X gate for compact qubit linking qubits $|C_{2}\rangle$and the target qubit $|T_{in}\rangle$ such that the target qubit is transformed as $X^{C_{2}}|T_{in}\rangle$. Likewise, is a two-qubit gate linking qubits $|C_{1}\rangle$and the target qubit $|T_{in}\rangle$ such that the target qubit is transformed as $X^{C_{1}}|T_{in}\rangle$. By substituting equation (S27) into equation (S26), we obtain

$\begin{matrix} G^{(1)}=\left(\begin{matrix} HTIT^{\dagger}ITIT^{\dagger}IH & O & O & O \\ O & HTIT^{\dagger}XTIT^{\dagger}XH & O & O \\ O & O & HTXT^{\dagger}ITXT^{\dagger}IH & O \\ O & O & O & HTXT^{\dagger}XTXT^{\dagger}XH \end{matrix}\right) \\ \text{ }=\left(\begin{matrix} I & O & O & O \\ O & I & O & O \\ O & O & I & O \\ O & O & O & -iX \end{matrix}\right). \end{matrix}$ (S18)

The input-out relation for partial circuit $G^{(2)}$ (enclosed by blue dashed line) in Fig. S6 is given by

$\begin{matrix} G^{(2)}=\left(T\otimes S\otimes I\right)\circ\left\{\left(\begin{matrix} I & O \\ O & X \end{matrix}\right)\otimes I\right\}\circ\left\{\left(\begin{matrix} T^{\dagger} & O \\ O & T^{\dagger} \end{matrix}\right)\otimes I\right\}\circ\left\{\left(\begin{matrix} I & O \\ O & X \end{matrix}\right)\otimes I\right\}\circ\left\{\left(\begin{matrix} T^{\dagger} & O \\ O & T^{\dagger} \end{matrix}\right)\otimes I\right\} \\ \text{ }=\left\{\left(\begin{matrix} 1 & 0 & 0 & 0 \\ 0 & i & 0 & 0 \\ 0 & 0 & e^{i\pi/4} & 0 \\ 0 & 0 & 0 & ie^{i\pi/4} \end{matrix}\right)\circ\left(\begin{matrix} IT^{\dagger}IT^{\dagger} & O \\ O & XT^{\dagger}XT^{\dagger} \end{matrix}\right)\right\}\otimes I \\ \text{ }=\left(\begin{matrix} 1 & 0 & 0 & 0 \\ 0 & 1 & 0 & 0 \\ 0 & 0 & 1 & 0 \\ 0 & 0 & 0 & i \end{matrix}\right)\otimes I \\ \text{ }=\left(\begin{matrix} I & O & O & O \\ O & I & O & O \\ O & O & I & O \\ O & O & O & iI \end{matrix}\right). \end{matrix}$ (S19)

It turns out that $G^{(2)}$is the same as $C^{2}(iI)$which can be written as

$G^{(2)}=C^{2}(iI)=I\otimes I\otimes I\left(|0\tilde{0}\rangle\langle0\tilde{0}|+|0\tilde{1}\rangle\langle0\tilde{1}|+|1\tilde{0}\rangle\langle1\tilde{0}|\right)+I\otimes I\otimes\left(iI\right)|1\tilde{1}\rangle\langle1\tilde{1}|$. (S20)

From equations (S18), (S19), we obtain

$C^{2}(X)=G^{(2)}\circ G^{(1)}=\left(\begin{matrix} I & O & O & O \\ O & I & O & O \\ O & O & I & O \\ O & O & O & X \end{matrix}\right),$ (S21)

which is a special case of (S22) for $U=X$and $m=2$.

In Fig. S6 a partial circuit $G^{(1)}$ corresponding to equations (S16)-S(18) is enclosed with the red dotted line. The corresponding QKM for $G^{(1)}$is shown below the quantum circuits. Here the superscript “*All”* in equations (S16) and (S17) denotes the case in which all the entries in QKM correspond to the same unitary operator. Likewise, the partial circuit $G^{(2)}$inside of the blue-dashed line corresponds to equations (S19) and (S20). The corresponding QKM for $G^{(2)}$ is shown below the quantum circuits. In the bottom of Fig. S6, the product of QKM for $G^{(1)}$ and $G^{(2)}$ is also depicted and shown to be corresponding to the representation of $C^{2}(X)$, as can be seen from the Fig. S2. In Fig. S6 we show the decomposition of $C^{2}(X)$circuits using the QKM as a combination of single qubit gates and $CNOT$ gates. This decomposition of $C^{2}(X)$circuit is well known,^3^ and therefore is a good example for demonstrating a usefulness of QKM.

**2. The Reduction of a 4-qubit Toffoli gate** $C^{3}(X)$

We first study the partial circuit A as illustrated in Fig. 4 of the main text. The target state $|T_{in}\rangle$ becomes

$|T_{out}\rangle=HTX^{C_{1}C_{2}}T^{\dagger}X^{C_{2}C_{3}}TX^{C_{1}C_{2}}T^{\dagger}X^{C_{2}C_{3}}H|T_{in}\rangle$. (S22)

It is straight forward to show that $|T_{out}\rangle=|T_{in}\rangle$ when $C_{1}C_{2}=0$ or $C_{1}C_{2}=1,\text{ }C_{3}=0$.

When $C_{1}=C_{2}=C_{3}=1$, we obtain

$\begin{matrix} |T_{out}\rangle=HTX^{C_{1}C_{2}}T^{\dagger}X^{C_{2}C_{3}}TX^{C_{1}C_{2}}T^{\dagger}X^{C_{2}C_{3}}H|T_{in}\rangle\\ \text{ }=HTXT^{\dagger}XTXT^{\dagger}XH|T_{in}\rangle\\ \text{ }=e^{-i\pi/2}HTTTTH|T_{in}\rangle\\ \text{ }=-iX|T_{in}\rangle, \end{matrix}$ (S23)

where we have used the relation

$XT^{\dagger}X=e^{-i\pi/4}T$, (S24)

and $HTTTTH=X$. (S25)

Equations (S34) and (S35) are easy to prove from the definition of single qubit gates (S24). This proves the entries of the QKM for a partial circuit A in Fig. 4.

The QKM for a partial circuit B can be obtained by the same method. The target state $|T_{in}\rangle$ becomes

$|T_{out}\rangle=HTX^{C_{1}C_{2}}T^{\dagger}X^{C_{3}}TX^{C_{1}C_{2}}T^{\dagger}X^{C_{3}}H|T_{in}\rangle$. (S26)

It is again straight forward to show that $|T_{out}\rangle=|T_{in}\rangle$ when $C_{1}C_{2}=0$ or $C_{1}C_{2}=1,\text{ }C_{3}=0$.

When $C_{1}=C_{2}=C_{3}=1$, we obtain

$\begin{matrix} |T_{out}\rangle=HTX^{C_{1}C_{2}}T^{\dagger}X^{C_{3}}TX^{C_{1}C_{2}}T^{\dagger}X^{C_{3}}H|T_{in}\rangle\\ \text{ }=HTXT^{\dagger}XTXT^{\dagger}XH|T_{in}\rangle\\ \text{ }=e^{-i\pi/2}HTTTTH|T_{in}\rangle\\ \text{ }=-iX|T_{in}\rangle, \end{matrix}$ (S27)

A comparison of the above equaitons shows that the reduced partial circuit B (S27) is obtained from the original partial circuit A (S23) by the replacement $X^{C_{2}C_{3}}\to X^{C_{3}}$.

In partial circuit C, $|C_{1}\rangle$ and $|T_{in}\rangle$ are not changed. The input states $|C_{2}\rangle$ and $|C_{3}\rangle$ are transformed to output states as

$|C_{2}\rangle\to T^{C_{1}}|C_{2}\rangle$, (S28)

and $|C_{3}\rangle\to SX^{C_{1}C_{2}}T^{\dagger}X^{C_{1}C_{2}}T^{\dagger}|C_{3}\rangle$. (S29)

When $C_{1}C_{2}=0$,

$SX^{C_{1}C_{2}}T^{\dagger}X^{C_{1}C_{2}}T^{\dagger}=ST^{\dagger}T^{\dagger}=I$ (S30)

and

$SX^{C_{1}C_{2}}T^{\dagger}X^{C_{1}C_{2}}T^{\dagger}=SXT^{\dagger}XT^{\dagger}=e^{-i\pi/4}S$ (S31)

when $C_{1}C_{2}=1$.

As a result, when $C_{1}=C_{2}=C_{3}=1$, we obtain

$|C_{2}\rangle\otimes|C_{3}\rangle\to\left(e^{i\pi/4}|C_{2}\rangle\right)\otimes\left(e^{-i\pi/4}S|C_{3}\rangle\right)=|C_{2}\rangle\otimes\left(i|C_{3}\rangle\right)$. (S32)

Here we have used the eigenstatesfor *S, where*

$S|0\rangle=|0\rangle,\text{ }S|1\rangle=i|1\rangle$. (S33)

Considering all the possible cases, we obtain QKM entries shown in Fig. 5 for the partial circuit C.

**4.** **The Reduction of a 5-qubit Toffoli gate** $C^{4}(X)$

We study a 5-qubit Toffoli gate as illustrated in Fig. S7. The target state $|T_{in}\rangle$ becomes

$|T_{out}\rangle=HTX^{C_{3}C_{4}}T^{\dagger}X^{C_{1}C_{2}}TX^{C_{3}C_{4}}T^{\dagger}X^{C_{1}C_{2}}H|T_{in}\rangle$. (S34)

It is straight forward to show that $|T_{out}\rangle=|T_{in}\rangle$ when $C_{1}C_{2}=0$or $C_{1}C_{2}=1,\text{ }C_{3}C_{4}=0$.

When $C_{1}C_{2}=C_{3}C_{4}=1$, we obtain

$\begin{matrix} |T_{out}\rangle=HTX^{C_{3}C_{4}}T^{\dagger}X^{C_{1}C_{2}}TX^{C_{3}C_{4}}T^{\dagger}X^{C_{1}C_{2}}H|T_{in}\rangle\\ \text{ }=HTXT^{\dagger}XTXT^{\dagger}XH|T_{in}\rangle\\ \text{ }=e^{-i\pi/2}HTTTTH|T_{in}\rangle\\ \text{ }=-iX|T_{in}\rangle, \end{matrix}$ (S35)

and

$|C_{4}'\rangle=S^{C_{1}C_{2}C_{3}}|C_{4}\rangle$ . (S36)

Therefore $|C_{4}'\rangle\otimes|T_{out}\rangle$ becomes

$|1\rangle\otimes\left(X|T_{in}\rangle\right)$ for $C_{1}C_{2}=C_{3}C_{4}=1$. (S37)

**Correspondence and requests for materials should be addressed to D. A. (e-mail:** [**dahn@uos.ac.kr**](mailto:dahn@uos.ac.kr)**)**

REFERENCES

1. Barenco, A., Bennett, C. H.,Cleve, R., DiVincenzo, D., Margolus, N., Shor, P., Sleator, T., Smolin, J. A. & Weinfurter, H. Elementary gates for quantum computation. *Phys. Rev. A* **52**, 3457-3467 (1995).
2. Ahn, D. Quantum Karnaugh map.US Patent 8,671,369 (2014).
3. Nielsen, M. A., Chuang,I. L. Quantum Computation and Quantum Information, (Cambridge University Press, 2000). pp. 172-202.
4. Krauss, B. & Cirac, J. I. Optimal creation of entanglement using a two-qubit gate. *Phys. Rev. A* **63**, 062309 (2001)
5. DiVincenzo, D., Two-bit gates are universal for quantum computation. *Phys. Rev. A* **51**, 1015 (1995).
6. Lanyon, B. P. *et al.* Simplifying quantum logic using higher-dimensional Hilbert spaces. *Nat. Phys.* **5**, 134 (2009).


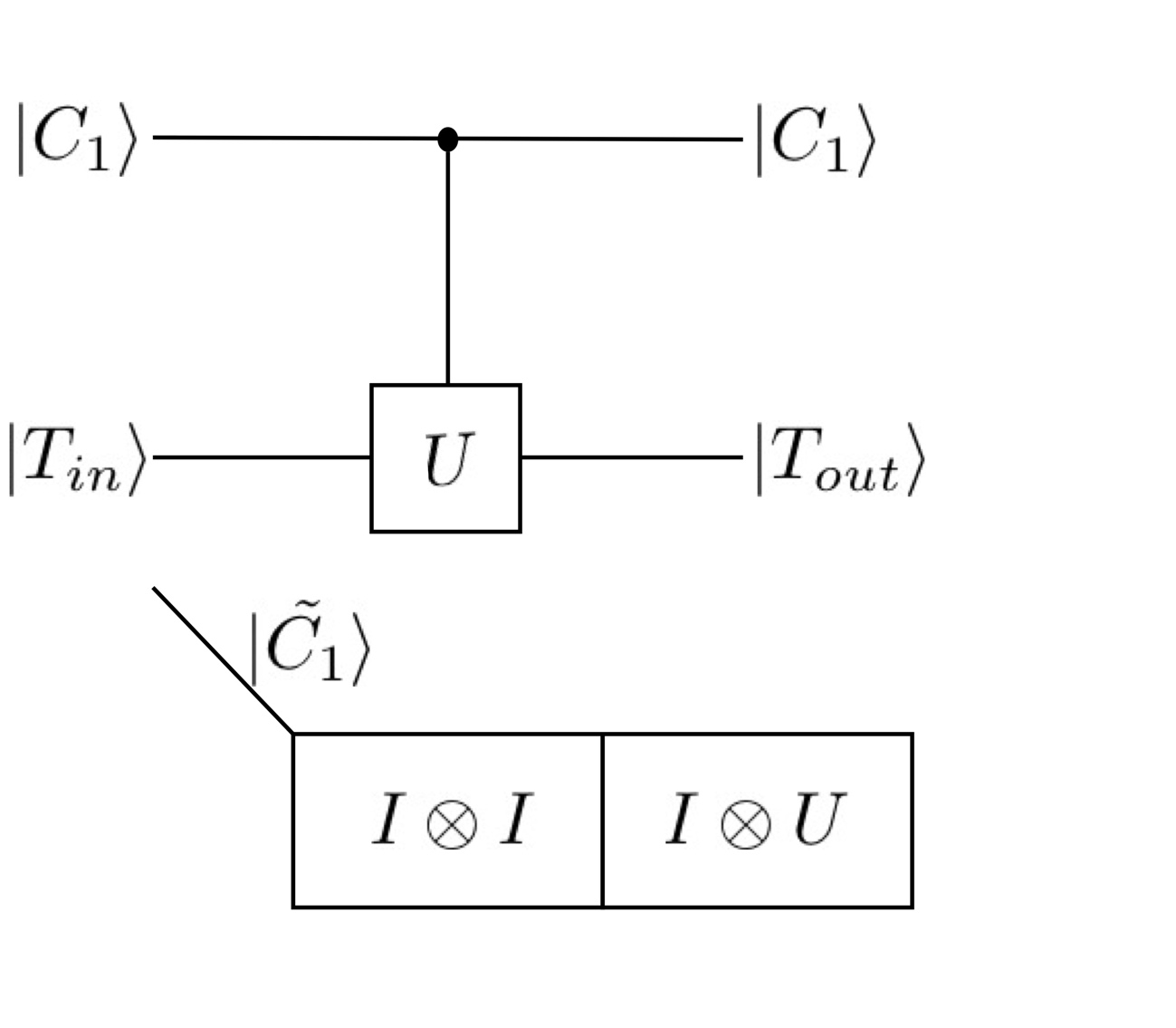


Figure S1. The quantum Karnaugh map (QKM) of the quantum circuit for a two-qubit controlled unitary gate based on equation (S12).


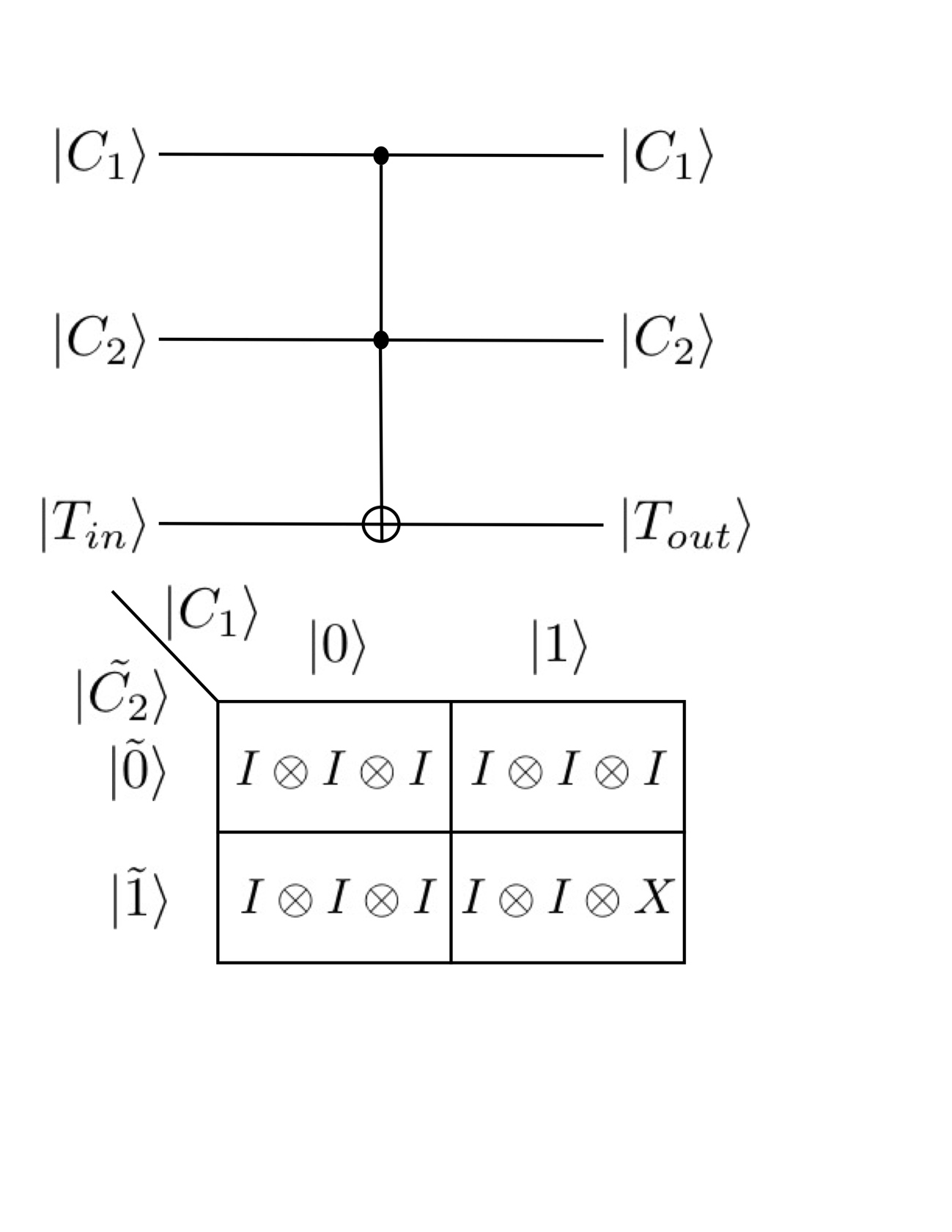


Figure S2. The 3-qubit Toffoli gate and the corresponding QKM.


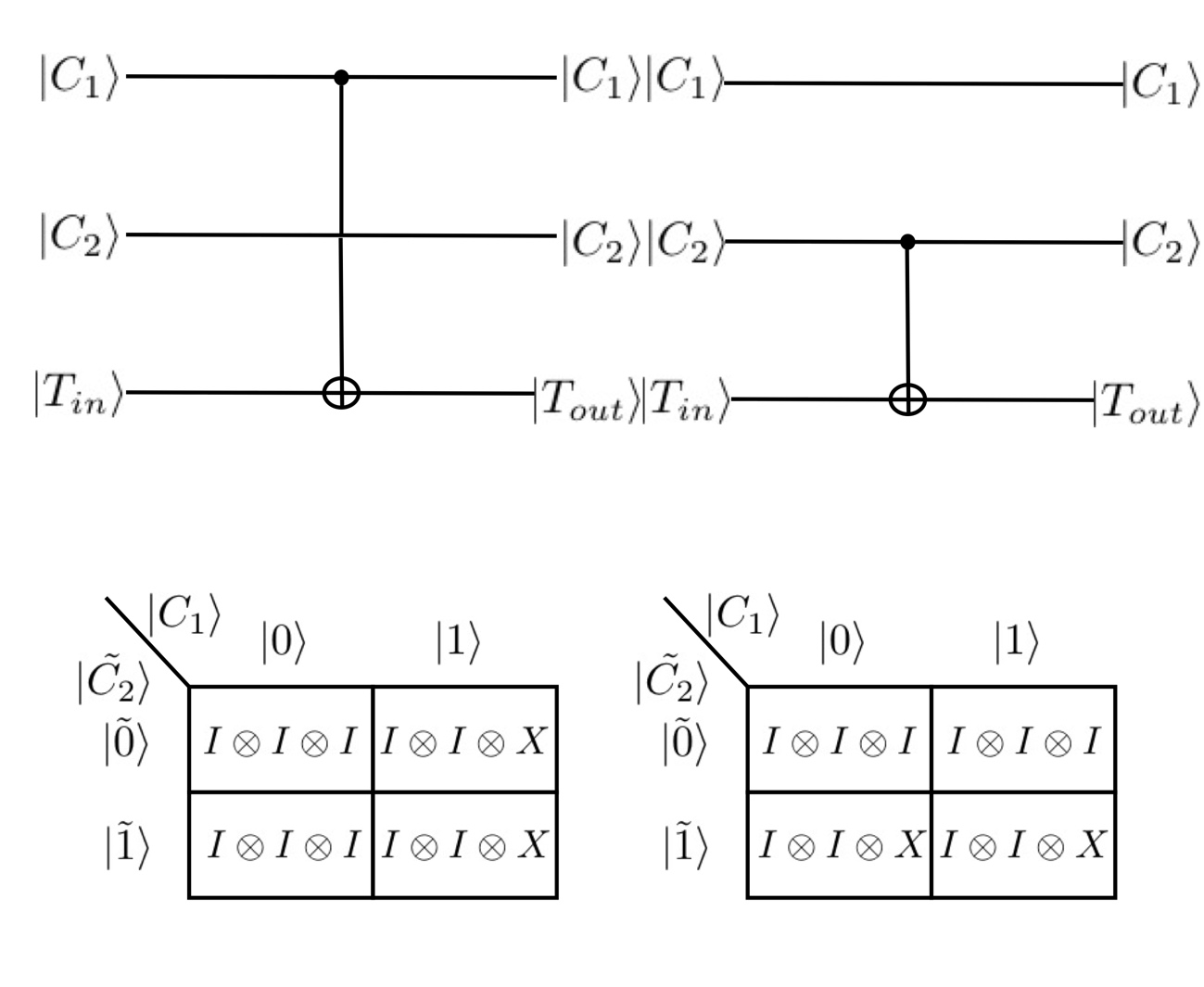


Figure S3. Controlled-NOT gates or $C^{1}(X)$ gates embedded in the 3-qubit quantum circuit and their corresponding QKMs.


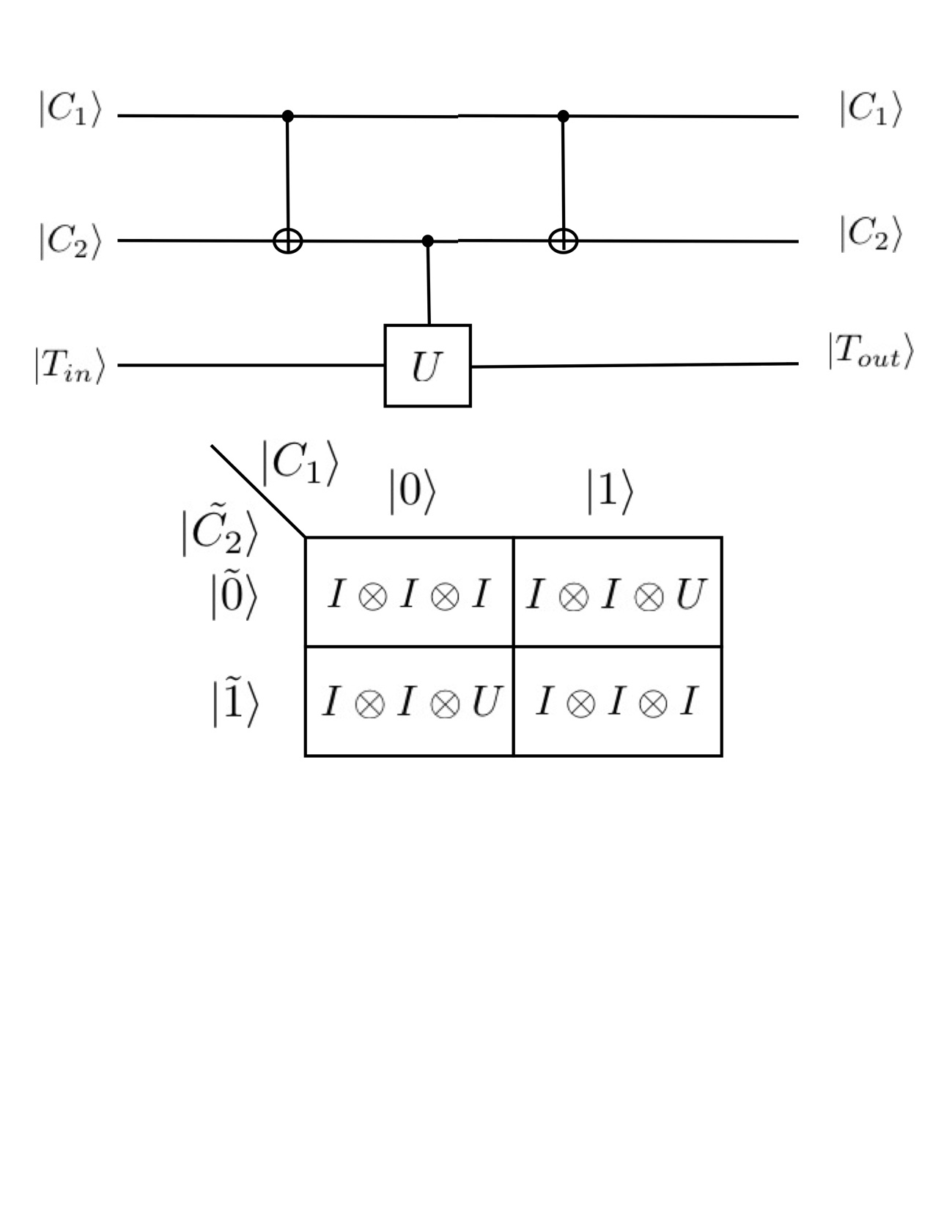


Figure S4. A quantum circuit $G(U)$,which executes a unitary operation $U$ when one of the control qubits is in the state$|1\rangle$, i.e., the value of the Exclusive-OR operation of the two control bits is 1, and the corresponding QKM which is constructed from $G(U)$ in the lower part of the figure.


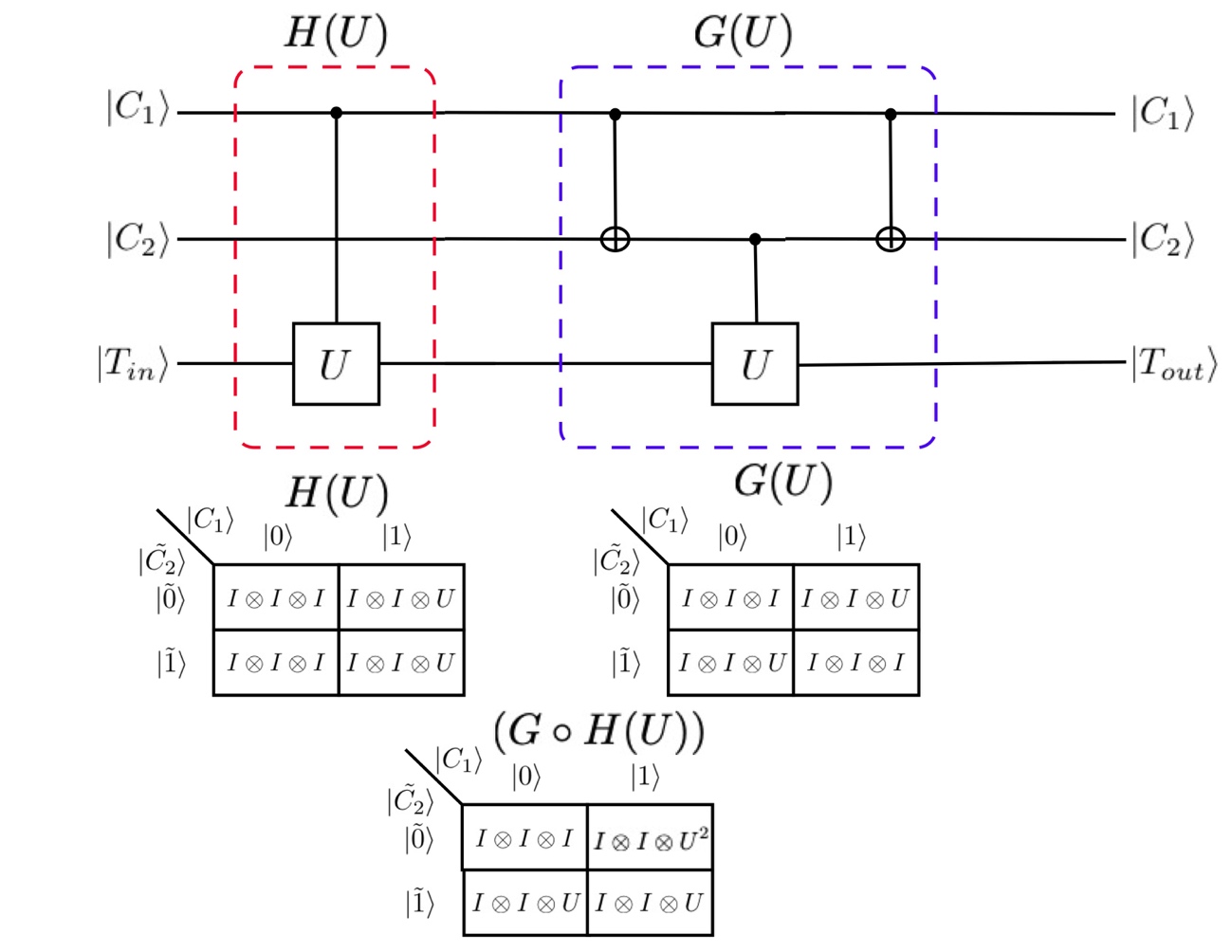


Figure S5. The representation of the 3-qubit quantum circuit with the combinational rule defined by equations (S18) and (S19) and the resulting QKMs.


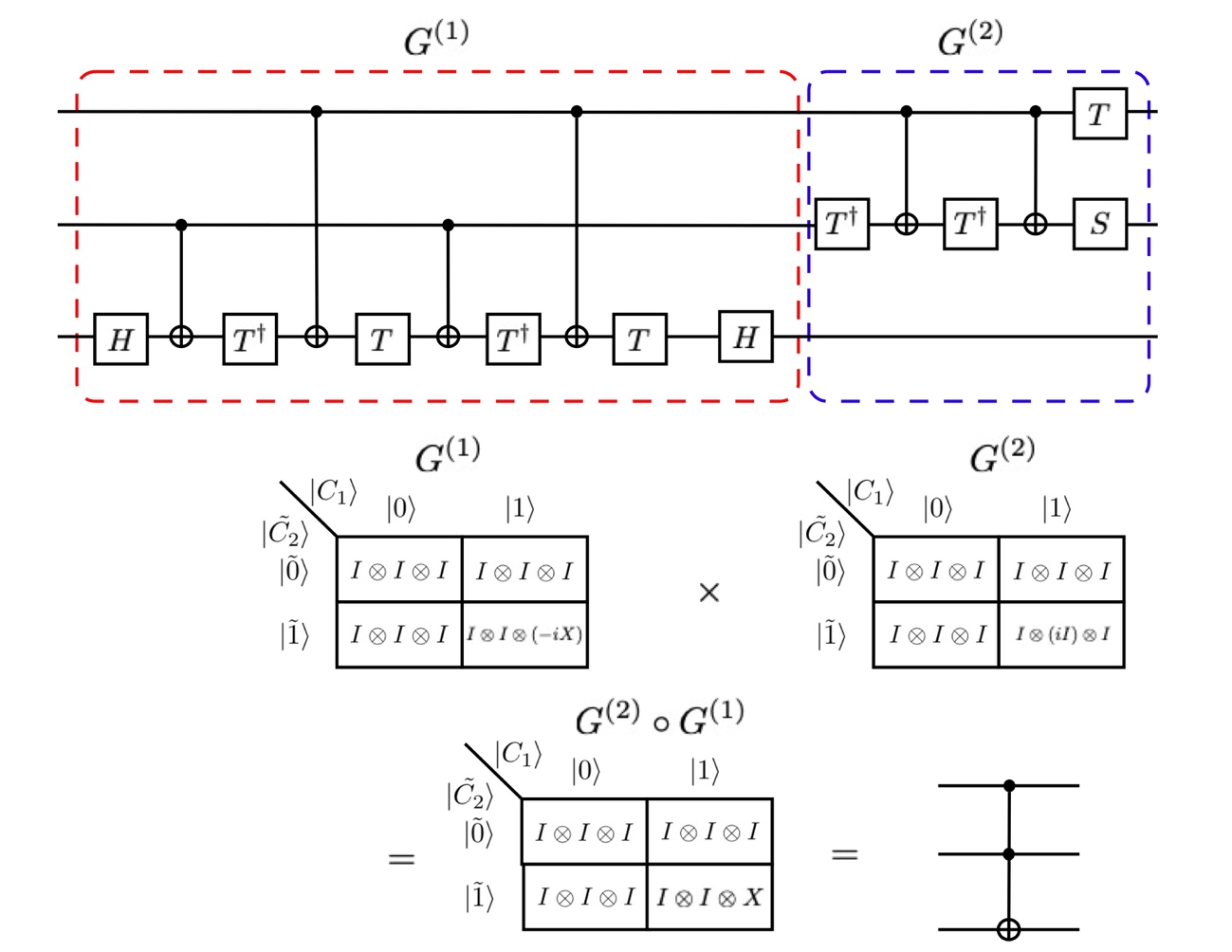


Figure S6. The elementary decomposition of $C^{2}(X)$ 3-qubit Toffoli gate and its QKM representation.


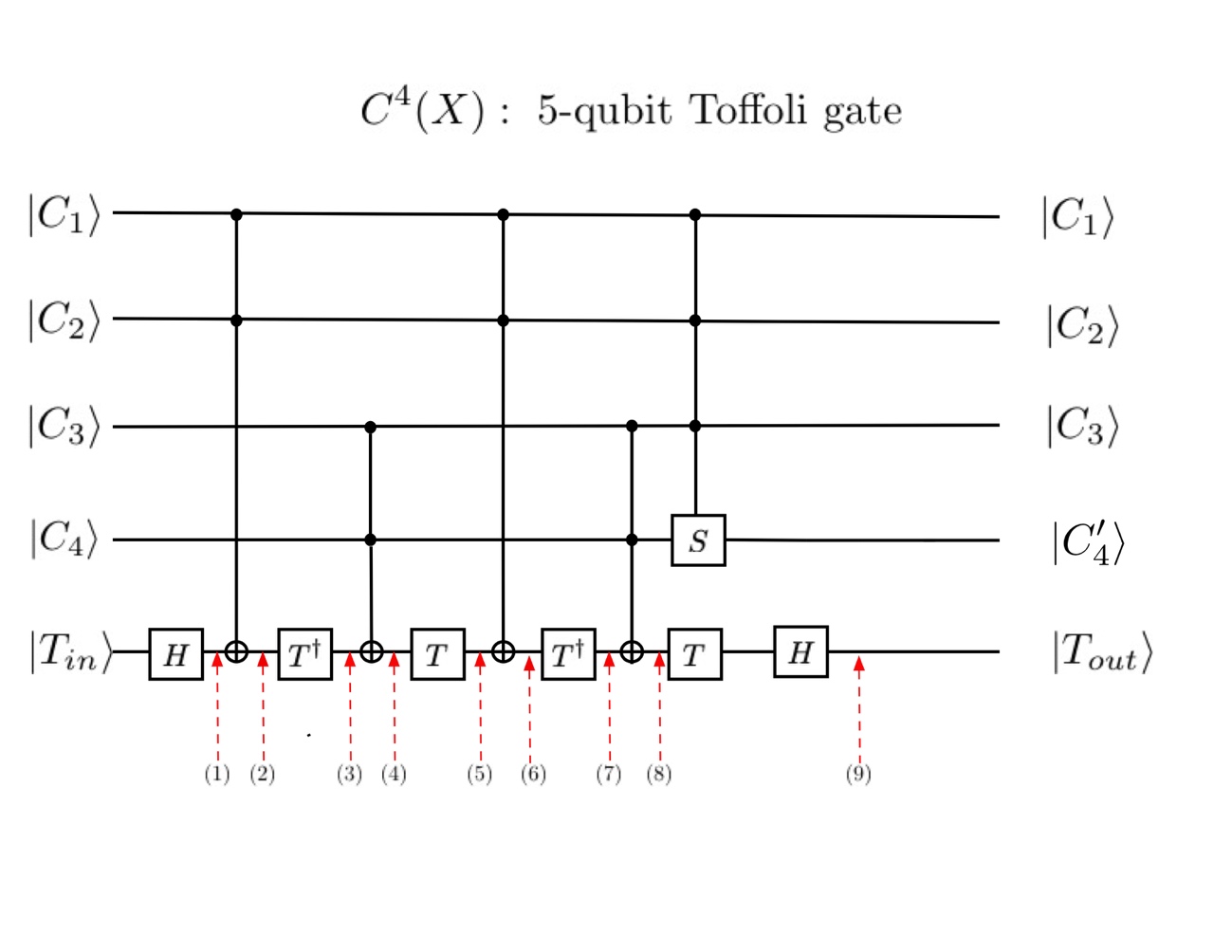


Figure S7 Minimum representation of 5-qubit Toffoli gate using QKM
